# A real-time forecasting framework for emerging infectious diseases affecting animal populations

**DOI:** 10.1371/journal.pcbi.1014716

**Published:** 2026-09-10

**Authors:** Meryl Theng, Simin Lee, Andrew C. Breed, Sharon Roche, Emily Sellens, Catherine Fraser, Kelly Wood, Chris P. Jewell, Mark A. Stevenson, Chris Baker, Simon M. Firestone

**Affiliations:** 1 School of Mathematics and Statistics, The University of Melbourne, Melbourne, Victoria, Australia; 2 Melbourne Centre for Data Science, The University of Melbourne, Melbourne, Victoria, Australia; 3 Melbourne Veterinary School, The University of Melbourne, Melbourne, Victoria, Australia; 4 Epidemiology, Surveillance and Laboratory Section, Australian Government Department of Agriculture, Fisheries and Forestry, Canberra, Australian Capital Territory, Australia; 5 School of Veterinary Science, University of Queensland, Brisbane, Queensland, Australia; 6 Biosecurity & Food Safety, New South Wales Department of Primary Industries, Orange, New South Wales, Australia; 7 Department of Mathematics and Statistics, Lancaster University, Bailrigg, Lancaster, United Kingdom; University of Zaragoza: Universidad de Zaragoza, SPAIN

## Abstract

Infectious disease forecasting has become increasingly important in public health. However, forecasting tools for emergency animal diseases, particularly those offering real-time decision support when parameters governing disease dynamics are unknown, remain limited. We introduce a generalised modelling framework for near-real-time forecasting of the temporal and spatial spread of infectious livestock diseases using data from the early stages of an outbreak. We applied the framework to the 2007 equine influenza outbreak in Australia, generating forecasts at three timepoints across four regional clusters. Prediction targets included future daily case counts, outbreak size, peak timing and duration, and spatial distributions of future spread. We evaluated how well the forecasts predicted daily cases and the spatial distribution of case counts, using skill scores (a measure of probabilistic forecast accuracy) as a benchmark for future model improvements. Forecast accuracy, certainty, and skill improved after formation of the outbreak’s peak, while early forecasts were more uncertain or prone to overestimation, highlighting the need for caution when interpreting pre-peak predictions, particularly when the impacts of control policies on future transmission are not adequately represented in the model. Spatial forecasts of broad, relative risk patterns were more robust than precise predictions of risk at the individual premises level or exact daily cases counts, supporting geographically targeted response strategies. Overall, this framework supports real-time decision-making in livestock disease outbreaks when applied with appropriate consideration of uncertainty, and establishes a foundation for future refinements and applications to other animal diseases.

## Author summary

Infectious disease outbreaks in livestock can spread rapidly, making timely and well-informed decisions critical for limiting their impact. However, predicting how an outbreak will evolve is difficult, especially early on when little is known about how the disease spreads. In this study, we developed a flexible modelling framework that uses data collected during the early stages of an outbreak to generate real-time forecasts of how disease may spread over time and space. We applied our approach to the 2007 equine influenza outbreak in Australia and assessed how well it could predict future cases and identify areas at higher risk. We found that forecasts became more accurate as more data became available, and that short-term predictions were generally more reliable than those made further into the future. Importantly, while precise predictions of case numbers or individual affected farms were often uncertain, the model was better at identifying “hotspot” areas of higher risk. These findings suggest the model is most useful for guiding targeted surveillance and control to these higher-risk zones, particularly when its uncertainty and assumptions are carefully considered.

## 1. Introduction

Rapidly spreading infectious animal disease outbreaks pose serious risks to food security, international trade, livelihoods, animal welfare, and public health. Major examples include the 2001 foot-and-mouth disease (FMD) epidemic in the UK [1], the ongoing global high pathogenicity avian influenza (HPAI) H5N1 clade 2.3.4.4b outbreak [2], 2007–2016 Q fever outbreak in the Netherlands [3], and the 2019–2021 lumpy skin disease (LSD) outbreaks in Asia [4]. Responding to these emergencies is challenging for animal health authorities and livestock industries, as they must make critical, time-sensitive decisions based on an uncertain future. To help address this, mathematical modelling has become an important tool for guiding outbreak responses, as shown during the COVID-19 human health pandemic, offering insights that can be applied in animal health crises.

A range of mathematical models have been developed to support outbreak preparedness and response, varying greatly in mechanistic detail and computational cost. On the simpler end of the spectrum, there are phenomenological approaches, such as the logistic growth model and the generalised Richards model, that make minimal assumptions about disease dynamics, and are typically used to rapidly estimate of key epidemiological parameters (e.g., epidemic growth rate, basic reproduction number) and generate quick short-term forecasts (e.g., [5,6]). Compartmental models such as SIR and SEIR introduce more mechanistic structure by explicitly representing stages of infection, yet remain relatively parsimonious and can be fitted to time‑series data to infer transmission dynamics and explore counterfactuals with modest computational cost [7,8]. At the more detailed end are large-scale, computationally intensive simulation frameworks, including spatially-explicit agent-based simulation models, such as AADIS [9], which aim to recreate outbreak behaviour in fine spatial and operational detail and are often used in longer-term strategic planning during “peacetime” to, for example, evaluate control strategies. Increasingly available computational power has expanded the types of models that can be subject to rigorous statistical analysis, particularly in the space between compartmental models and large-scale simulation frameworks. Fitting time-series data to generative models that incorporate greater mechanistic detail and spatial structure has enabled predictions at finer resolutions and evaluation of potential intervention strategies [10,11], capabilities that simpler models generally lack and that have been critical for decision-support during recent public health crises (e.g., [12,13]).

While data-driven, mechanistic models for real-time forecasting are well-established and rapidly advancing in public health, similar tools for animal health are relatively limited and untested. A common modelling approach in animal health uses detailed spatially-explicit simulators that represent the full range of outbreak dynamics (e.g., transmission pathways, host behaviours, and control measures) and consequently require the specification of many parameters, either fixed or sampled from distributions based on the published literature and expert opinion (e.g., AADIS, InterSpread; [14]). In contrast, simpler versions of these spatially‑explicit simulators are formulated with far fewer parameters, many of which are treated as unknown and inferred directly from the data through a statistical fitting process. Jewell et al. [15] made initial developments into this area with a full Bayesian Markov chain Monte Carlo (MCMC) approach to infer the underlying outbreak process and probability of undetected infections at premises during the 2007 UK outbreak of FMD. Subsequent studies have adapted this method and include applications for: retrospective estimation of epidemiological parameters for the 2007 equine influenza (EI) outbreak in Australia [16]; elucidation of spatiotemporal patterns of the HPAI H5N1 outbreak in the Mekong River Delta [17]; and comparison between real-time and retrospective evaluation of control policies for the 2007 and 2010 FMD outbreaks in the UK and Miyazaki, Japan [18].

Despite the potential of Bayesian MCMC approaches for generating spatiotemporal risk predictions and forecasts, several limitations exist [15]. First, MCMC is a serial algorithm that cannot be trivially parallelised to enhance performance, making runtime a major issue. Second, we currently lack software to efficiently specify and implement such epidemic models, requiring new training algorithms for each structural change to the model. Third, since most transition events (i.e., from susceptible to exposed states) are unobserved, we must treat transition timings as a large, highly correlated, latent data space. This slows mixing and demands more efficient sampling methods to estimate the posterior distribution accurately, but existing approaches often lack the operational speed and adaptability needed for real-time use, particularly the ability to rapidly re-specify and refit models as data, policies or disease characteristics evolve. These gaps highlight the need for more adaptable Bayesian modelling frameworks that can quickly modify or compare different models during an outbreak without the need to recode likelihood functions [19]. Addressing these limitations requires developing flexible and efficient approaches, alongside rigorous evaluation of their forecasting capabilities.

Here, we introduce a generalised Bayesian modelling framework that produces near-real-time forecasts of temporal and spatial disease spread using data available in the first few weeks of a livestock disease outbreak and demonstrate how its predictions can be used to support decision-making during that period. We assume that existing premises- and herd-level data (location details, premises area and number of animals) are available prior to an outbreak, as we use this information to model how infections spread within and between these properties. As an outbreak progresses, we receive updated information on confirmed infectious premises (i.e., outbreak surveillance data), and this time series data is used to estimate parameters in our model using a likelihood-free Bayesian approach. Forward simulations (i.e., from the date of observation) generated from these parameter estimates represent the forecasts from the model. We apply our modelling framework on time slices of the dataset for the largest clusters of the 2007 EI outbreak in Australia, with the view of extending it to higher-priority, more complex emergency livestock diseases (e.g., FMD, LSD). We evaluate the model’s performance on this dataset by measuring forecast skill relative to a naive benchmark forecast, and in doing so, establish a baseline from which future extensions and improvements can be developed and evaluated against.

## 2. Methods

**Fig 1:**
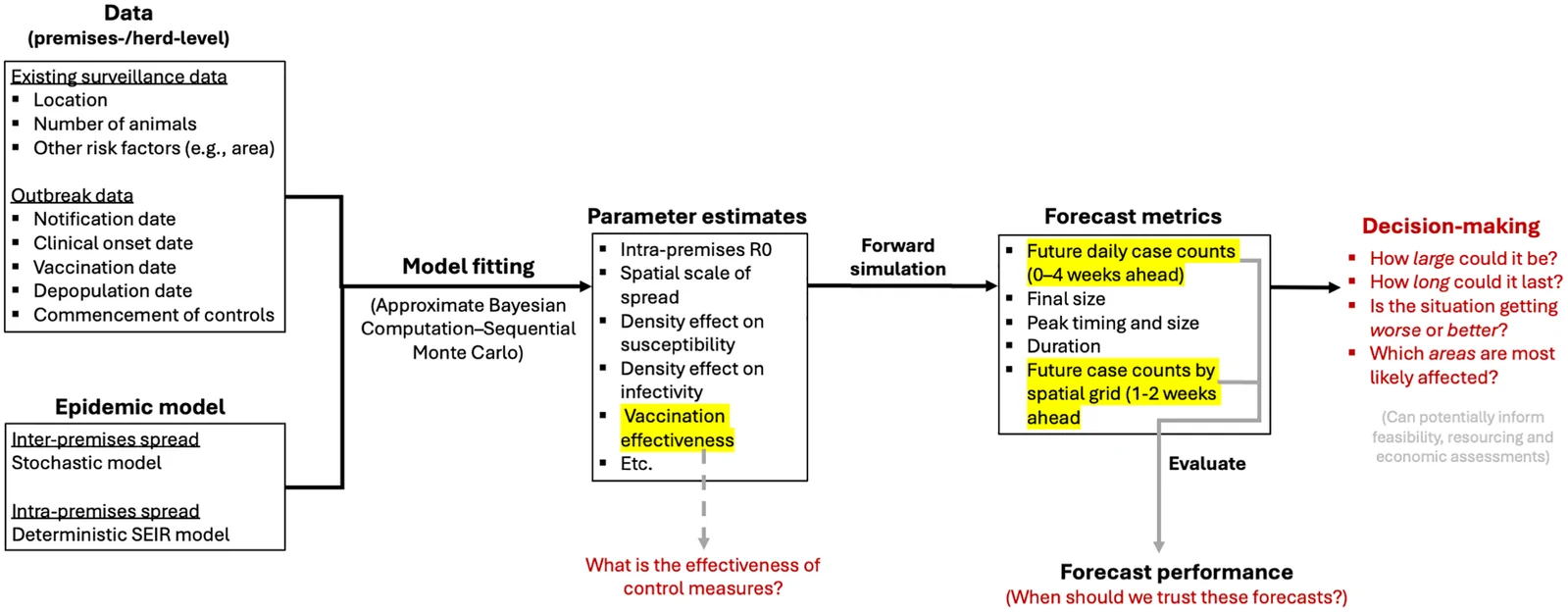
Conceptual representation of our modelling framework for real-time forecasting of a livestock disease outbreak. Model outputs can directly address questions (in red) to support decision-making, potentially informing feasibility, resource allocation, and economic assessments.

First, we discuss a generic modelling framework applicable to a broad range of animal disease epidemics (Fig 1).

We begin by describing the epidemic model in Section 2.1, which comprises: (i) a stochastic model of *inter*-premises disease transmission influenced by premises area, number of animals and distance to other premises; and (ii) a deterministic SEIR model of *intra*-premises transmission between animals. We then outline an efficient approximate Bayesian computation (ABC) approach, in which we accept the simulations (i.e., particles) that are sufficiently matched to the observed data available at a given point in time (in Section 2.2). In Section 2.3, we characterise forward projections of the accepted particles (i.e., data simulated beyond the timepoint) with five forecast targets – (i) future daily case counts, (ii) final size of outbreak, (iii) peak timing and size, (iv) duration of outbreak, (v) spatial pattern of infection risk, (vi) future case counts on a spatial grid – that focus on addressing the questions: “How large could the outbreak get?”; “Is the situation getting worse or better?”; and “Which areas/premises are most likely affected?”. We then detail an evaluation approach that measures how accurately a forecast predicted future daily case counts and future case counts by spatial grid relative to a naive benchmark forecast (future values equal the last observed value), which addresses the question “When should we trust these forecasts?” (in Section 2.3).

To illustrate our methodology, we generated predictions by applying our framework to premises-level data from the 2007 equine influenza outbreak in Australia (in Section 2.4). We observed the epidemic at three time points (3, 5 and 7 weeks after the outbreak was first detected; Table 1), using the data available to produce forecasts for four of the largest outbreak clusters (Sydney Basin, Central-Coast-Maitland-Newcastle, Tamworth and Hunter Valley; see Section 2.4.1 for background). We show how forecast targets, parameter estimates and forecast performance refine as the epidemic progresses and how they vary between clusters.

**Table 1:** Descriptive statistics for highly affected regions (or clusters) in the Australian 2007 equine influenza outbreak, used as study sites in the development and validation of a real-time model of equine influenza in Australia.

	Sydney	CCMN	Tamworth	Hunter Valley	All outbreak affected areas
Number of horse premises	7387	6549	2317	1522	103,221
Number of infected premises	2875	1293	582	404	8858
Date of first detection	25 August 2007	26 August 2007	26 August 2007	28 August 2007	24 August 2007

### 2.1 Epidemic model

The basis for forecasting is a stochastic model of the transmission mechanism between individual livestock premises (adapted from [15,16]). Hence, the unit of interest is at the premises level rather the individual animal. We make two key assumptions in the model.

Firstly, we assume that out of a total population size of N premises, each premises is in one of four states at any given time point t:

- **S**usceptible premises are disease-free and contain no infected animals but are able to be infected;
- **E**xposed premises contain animals that have been infected but are not yet infectious to other premises;
- **I**nfectious premises contain infected animals capable of transmitting the disease to susceptible premises;
- **R**ecovered (or removed) premises contain animals that have either recovered from the disease or been culled, and therefore play no further part in the epidemic.

The only transitions between states allowed are: from susceptible to exposed, from exposed to infectious, and from infectious to recovered (or removed). Each infectious premises k was associated with event (i.e., transition) times $t_{e,k}$, $t_{i,k}$ and $t_{r,k}$ representing the time of exposure, onset of infectiousness and recovery, respectively. Since these times are not (or tend not to) be directly observable, we have to infer them from observable data, such as time of onset of clinical signs, time of notification (to the appropriate animal health authority) and time of removal or sanitary measures (intended to render the premises no longer infectious; see Section 2.1.2 for details).

Secondly, we allow for time-varying infectivity of premises, corresponding to intra-premises infection of more and more animals over time [15]. We model intra-premises infectivity using a deterministic SEIR compartmental model based on the number of animals recorded as present on each premises at the start of the outbreak (described in Section 2.1.2). The intra-premises transmission model is used to determine a premises’ infectious periods (i.e., recovery time) and the prevalence of infectious animals at time t, which contributes to premises-to-premises infection pressure (see Section 2.1.2).

#### 2.1.1 Transmission (inter-premises).

The inter-premises model is driven by a spatiotemporal inhomogeneous Poisson process, simulated with daily time-steps, using a tau-leap algorithm [15,20]. Essentially, this is a spatially and temporally explicit modelling process that allows for different rates of new exposures to occur over time and space driven by the infection pressure in the system.

The Poisson point process is governed by the overall disease transmission rate, $\tau_{t}$, which is the sum of the individual infection pressures $\beta_{jk}(t)$ (Eq. 1) acting on all susceptible premises j from all infectious premises k at time t:


$$\begin{array}{c} \tau (t)=\alpha +\sum_{j\in S_{t},k\in I_{t}}\beta_{jk}(t) \end{array}$$

where α is the background transmission rate representing transmission not explicitly modelled (e.g., new seeding events from outside the studied cluster, such as repeated re-introduction through contact-network transmission from other regions).

Infectious pressure exerted by each pair of individual infectious and susceptible premises $\beta_{jk}(t)$ (Eq. 1) is modulated by factors which influence the susceptibility and infectivity of each premises, including numbers of animals and how close they are to each other, giving:


$$\begin{array}{c} \beta_{ij}(\text{t})=\beta_{\text{0}}\times q_{k}(\text{t})\times s_{j}(\text{t})\times K(j,k)\; \end{array}$$ (1)

where $\beta_{\text{0}}$ is the baseline transmission rate, $q_{k}(t)$ is the infectivity of infectious premises k at time t (Eq. 2), $s_{j}(t)$ is the susceptibility of susceptible premises j at time t (Eq. 3), and K(j,k) is a spatial kernel describing the transmission rate decay with increasing Euclidean distance between k and j. The latter three functions can be formulated specific to each disease scenario and may be easily modified.

Here, we describe a single-species formulation and define the three functions as follows:

First, the infectivity of premises k at time t is defined as,


$$\begin{array}{c} q_{k}(\text{t})={\left(\frac{n_{k}}{a_{k}}\right)}^{\zeta }\times h_{k}(t) \end{array}$$ (2)

where $n_{k}$ and $a_{k}$ are the number of animals on, and area of infectious premises k, respectively, and ζ is an unknown parameter that allows for the non-linear effect of density of animals on infectivity. $h_{k}(t)$ is the proportion of infective individuals within premises k at time t (i.e., prevalence), obtained directly from the solution of an SEIR system of differential equations which we describe in Section 2.1.2.

Second, the susceptibility of premises j at time t is defined as,


$$\begin{array}{c} s_{j}(\text{t})={\left(\frac{n_{j}}{a_{j}}\right)}^{\xi }\times \;\omega (\text{t}) \end{array}$$ (3)

where $n_{j}$ and $a_{j}$ are the number of animals on, and area of infectious premises j, respectively, and ξ is an unknown parameter that allows for the non-linear effect of density of animals on susceptibility. The additional term, ω(t), represents the effect of control measures implemented to reduce susceptibility, such as indoor housing of animals or vaccination (see Section 2.4.2 for the formulation used in the case study).

Finally, the spatial transmission kernel K(j,k), which captures how transmission rate decays with Euclidean distance between premises j and k, can be selected from a range of decay function options, including the power-law, exponential, geometric or Cauchy forms. The choice of kernel may be guided pragmatically by computational considerations or empirically by selecting the best-fitting form based on the data [21,22]. The formulation used in the equine influenza case study is described in Section 2.4.2.

#### 2.1.2 Infectious periods and prevalence (intra-premises).

The following differential equations model the epidemic within each premises (herd, or other unit of interest); for each newly infectious premises k at the time that it is simulated to be exposed $t_{e,k}$ we initialise an SEIR model with an (unobserved) initial Exposed count (e) sampled from a uniform distribution U(1,n).


$$\begin{array}{c} \frac{ds}{dt}=-\frac{\beta_{\text{intra}}s(t)i(t)}{n}, \end{array}$$

$$\begin{array}{c} \frac{de}{dt}=\frac{\beta_{\text{intra}}s(t)i(t)}{n}-\sigma_{\text{intra}}e(t), \end{array}$$

$$\begin{array}{c} \frac{di}{dt}=\sigma_{\text{intra}}e(t)-\gamma_{\text{intra}}i(t), \end{array}$$

$$\begin{array}{c} \frac{dr}{dt}=\gamma_{\text{intra}}i(t). \end{array}$$

$\beta_{\text{intra}}$, $\sigma_{\text{intra}}$, and $\gamma_{\text{intra}}$ represent the transition rates between states, and the counts of animals in each state at time t are represented by s(t), e(t), i(t) and r(t) (totalling to n).

The prevalence of infectious individuals within a premises at time t, $h_{k}(t)=\frac{e_{k}(t)}{n_{k}}$, contributes to the calculation of infection pressures and the thinning of the Poisson process in the inter-premises model (see Appendix A in S1 File). Each newly infectious premises, k, is assumed to recover at time $t_{r}$ when there is <0.5 infections animals on the premises. Assumptions about the timing of onset of infectiousness ($t_{i,k}$) and onset of clinical signs ($t_{c,k}$) are scenario-specific and typically involve sampling from Gamma or Weibull distributions based on published details of the latent period ($t_{i,k}-t_{e,k}$), incubation period ($t_{c,k}-t_{e,k}$) and infectious periods ($t_{r,k}-t_{i,k}$). Delay from onset of clinical signs to timing of notification ($t_{n,k}-t_{c,k}$), and delay from notification to timing of depopulation ($t_{d,k}-t_{n,k}$, where applicable) may be fixed at a single value, entered as a distribution to be stochastically sampled or or directly inputted for premises in specific regions at specific times. See Section 2.4.2 for details of the assumptions used for our case study.

### 2.2 Model fitting (ABC-SMC)

The reliability of forecasts generated by a model depends on how reasonably it was parameterised. Bayesian approaches are widely used for model-based statistical inference of unknown parameters in stochastic epidemic models (e.g., [15,23,24]). More recently, approximate Bayesian computation (ABC) methods have gained traction in infectious disease modelling (e.g., [25]). Unlike MCMC methods, ABC approximates parameter posterior distributions without likelihood functions, which can be intractable or computationally costly to evaluate [26]. The flexibility of ABC approaches provides an attractive way to fit models to often incomplete and/or complex data in an accurate and timely manner.

We fit model outputs to observed data (daily case counts and total grid-cell case counts), using ABC with a particle filter algorithm called sequential Monte Carlo (ABC-SMC) [26]. Like all ABC methods, this involves sampling a candidate set of parameter values, $\theta^{*}$ from the prior distributions P(θ), and using this to simulate data from a model ($D^{*}$), in this case representing a time series of daily case counts and total grid-cell case counts. These are compared to the observed data D by using a distance function $d\left(D,D^{*}\right)$, and the candidate values satisfying $d\left(D,D^{*}\right)<\epsilon$ for some threshold ϵ are ‘accepted’. This process, known as ABC rejection sampling, repeats until $N_{\text{px}}$ ‘particles’ (i.e., parameter sets) are accepted, providing a set of particles that form posterior distributions of model parameters. However, naive rejection sampling is very inefficient because it explores the entire parameter space. ABC-SMC improves efficiency by propagating the set of particles through a sequence of intermediate distributions by gradually decreasing tolerance $\epsilon_{\text{1}}>\epsilon_{\text{2}}>...,>\epsilon_{G}$. Each intermediate distribution (called a generation) is obtained as a weighted sample from the previous distribution that has been perturbed through a kernel $K\left(\theta |\theta^{*}\right)$.

We adapt an ABC-SMC algorithm described by Minter and Retkute [25] to our two distance (or discrepancy) functions, one temporal and one spatial. To account for reporting delays, we only consider data starting from the day of first detection in a cluster ($t_{\text{detect}}=$ earliest notification) and ending at the timepoint minus some number of days $T=t_{\text{today}}-t_{\text{lag}}$.

The temporal distance function is the sum of absolute differences of daily case counts (number of exposed premises per day $E_{t}$) between the observed and simulated data,


$$\begin{array}{c} d_{\text{temp}}\left(D,D^{*}\right)=\sum_{t=t_{\text{detect}}}^{T}∥E_{t}-E_{t}^{*}∥, \end{array}$$

for $t=t_{\text{detect}},...,T$.

The spatial discrepancy measure is the Pearson’s correlation coefficient between the grid-specific total case counts ($E=\left(E_{\text{1}},E_{\text{2}},...,E_{X}\right)$) of observed and simulated data for $t=t_{\text{detect}},...,T$,


$$\begin{array}{c} d_{\text{spat}}\left(D,D^{*}\right)=\rho \left(E,E^{*}\right). \end{array}$$

Here, the spatial extent of the outbreak cluster is represented by X=X×X grid cells, and grid cells that do not contain premises are excluded.

We accept $\theta^{*}$ if the temporal distance is less than or equals to the temporal tolerance ($d_{\text{temp}}\left(D,D^{*}\right)\leq \epsilon_{\text{temp},g}$) *and* the spatial discrepancy is more than or equals to the spatial tolerance for generation g ($d_{\text{temp}}\left(D,D^{*}\right)\geq \epsilon_{\text{spat},g}$).

We used a normal distribution as our perturbation kernel following Beaumont [26], where $K\left(\theta |\theta^{*}\right)=\text{N}\left(\theta^{*},\sqrt{\text{2}\tau_{g-\text{1}}^{\text{2}}}\right)$, and the weight denominator for g>1 is $\sum_{i=\text{1}}^{N_{\text{px}}}w_{g-\text{1}}\cdot {\text{𝒻}}_{N}\left(\frac{\theta_{g}^{\left(i\right)}-\theta_{g-\text{1}}}{\sqrt{\text{2}\tau_{g-\text{1}}^{\text{2}}}}|\mu =\text{1},\sigma =\text{0}\right)$, where ${\text{𝒻}}_{N}$ is the probability density for the normal distribution and $\tau_{g-\text{1}}^{\text{2}}$ is the weighted empirical variance of the $\theta_{g-\text{1}}^{\left(i\right)}$s.

Details of the ABC-SMC algorithm used are provided in Appendix A in S1 File.

### 2.3 Forecast targets and evaluation

Forecast targets are the outcomes being predicted. Our model provides the following targets:

i. **future daily case counts**: daily case counts (exposed number of premises per day, $E_{t}$) for the 3 days prior to the timepoint, for the timepoint itself, and for the 4 weeks (28 days) after the timepoint
ii. **final size**: total case count over the course of the entire outbreak
iii. **peak timing and size**: day with the maximum case count and the case count for that day
iv. **duration**: first day to the last day (with a non-zero case count) of the outbreak
v. **spatial pattern of infection risk** (1–2 weeks ahead): the risk that a given premises becomes infectious during the current outbreak, presented as a map; risk was estimated by the proportion of simulations (out of 1000) in which a presently susceptible premises was infectious between the date of first detection and (a) 1-week ahead from the current date, and (b) 2- weeks from the current date.
vi. **spatial distribution of future case counts** (1- and 2- weeks ahead): spatial grid-cell case counts (exposed number of premises per cell, $E_{x}$) between the 3 days prior to the timepoint and 1- or 2-weeks ahead from the timepoint.

Forecast performance of the first and last target (i.e., future daily case counts, future grid-cell case counts) was quantified using Continuous Ranked Probability Scores (CRPS). CRPS is a widely-used metric for probabilistic forecasts [27]. It penalises forecasts for placing probability mass at a distance from the ground truth, with values increasing as more probability mass is allocated to incorrect values, and as the distance between these incorrect values and the ground truth increases.

For the future daily case counts, we calculated CRPS values for each $y_{\eta }$ observation of cases on day η, given observations $y_{\text{0}:t}$ for days 0…t (inclusive; as per [28]):


$$\begin{array}{c} P\left(Y_{\eta }\leq y\;\right|\;y_{\text{0}:t})=\;\sum_{i=\text{1}}^{N_{px}}H\left(y-{\tilde{y}}_{\eta }^{i}\right)\; \end{array}$$

$$\begin{array}{c} \text{CRPS}(Y_{\eta }|\;y_{\text{0}:t})=\sum_{y=\text{0}}^{Y_{\text{max}}}\left[P\left(Y_{\eta }\leq y\;\right|\;y_{\text{0}:t}\right)-\;H\left(y-y_{\eta }\right){]}^{\text{2}}\; \end{array}$$

$$\begin{array}{c} H(x)=\left\{ \begin{array}{cc} \text{1},\; & x\geq \text{0}\\ \text{0},\; & \text{otherwise} \end{array}\right., \end{array}$$

where ${\tilde{y}}_{\eta }^{i}$ is the simulated case count on day η from each particle i in the ensemble, and H denotes the Heaviside step function. The CRPS essentially calculates the difference between the forecast CDF (i.e., the probability that the actual cases will be less than or equal to a value y; $P\left(Y_{\eta }\leq y\;\right|\;y_{\text{0}:t})$) and the ‘perfect’ CDF from the actual observation $H\left(y-y_{\eta }\right)$.

We then compared the CRPS performance of our model M to that of a naive benchmark forecast N, which was simply the most recently observed value:


$$\begin{array}{c} Y_{t+h}=y_{t}, \end{array}$$

for h∈{−3−2,...,27,28}.

Forecast skill was calculated using the CRPS values for the model predictions (${\text{CRPS}}_{M}$) and that of the naive benchmark forecast (${\text{CRPS}}_{N}$):


$$\begin{array}{c} \text{skill}\left(M\right)=\frac{{\text{CRPS}}_{N}-{\text{CRPS}}_{M}}{{\text{CRPS}}_{N}}. \end{array}$$

Negative skill scores indicate that the forecasts performed worse than the naive benchmark, zero indicates that the forecasts performed as well as the naive benchmark, and positive values indicate that the forecasts performed better than the naive benchmark. The maximum possible skill score is one, which indicates that a forecast was perfectly accurate with no uncertainty.

Similarly, for future grid-cell case counts, we calculated CRPS values for each observation of total case counts within a spatial grid cell g for two periods: 1-week ahead (lead time of -3–7 days) and 2-weeks ahead (lead time of -3–14 days). Our naive benchmark forecast was the observed value for the most recent period of the same length:


$$\begin{array}{c} Y_{g,t-\text{2}:t+h}=y_{g,t-(\text{2}+h):t-\text{3}}, \end{array}$$

for h=7 or 14.

### 2.4 Application: 2007 Australian equine influenza outbreak

#### 2.4.1 Background.

Equine influenza is a highly contagious respiratory disease of horses. Australia experienced its first ever EI outbreak in August 2007, first detected on 24 August at the Centennial Park Equestrian Centre (CPEC) in Sydney. The outbreak spread rapidly through a largely unvaccinated horse population across two states (New South Wales and Queendland), infecting a reported total of 67,000 horses on 9,359 premises in less than 4 months [29]. Eradication was achieved in less than five months, through a combination of state-wide horse movement standstills following first detection, temporary ban of all horse events, on-premises biosecurity measures and targeted vaccination [30,31]. The measures, together with compensation payments, cost an estimated AUD 571 million and caused significant disruption to the horse industry [32]. Since the outbreak, numerous epidemiological analyses and curated datasets have become available [16,29,33], making the 2007 EI outbreak a well-documented and ideal case study for simulating real-time forecasting and evaluating its reliability for decision-support during animal-health emergencies.

To analyse this epidemic, we used a dataset that was prepared from premises-level covariate and contact tracing data provided by the New South Wales (NSW) and Queensland (QLD) State governments (data collation and cleaning details described in [16,33,34]). These data contained the following covariates: address, geographical location (longitude and latitude of the centroid of the premises), number of horses present, premises area, vaccination status, and vaccination date for NSW data. Infectious premises data also included the estimated date of onset of first clinical signs of the first horse affected (‘onset date’).

**Fig 2:**
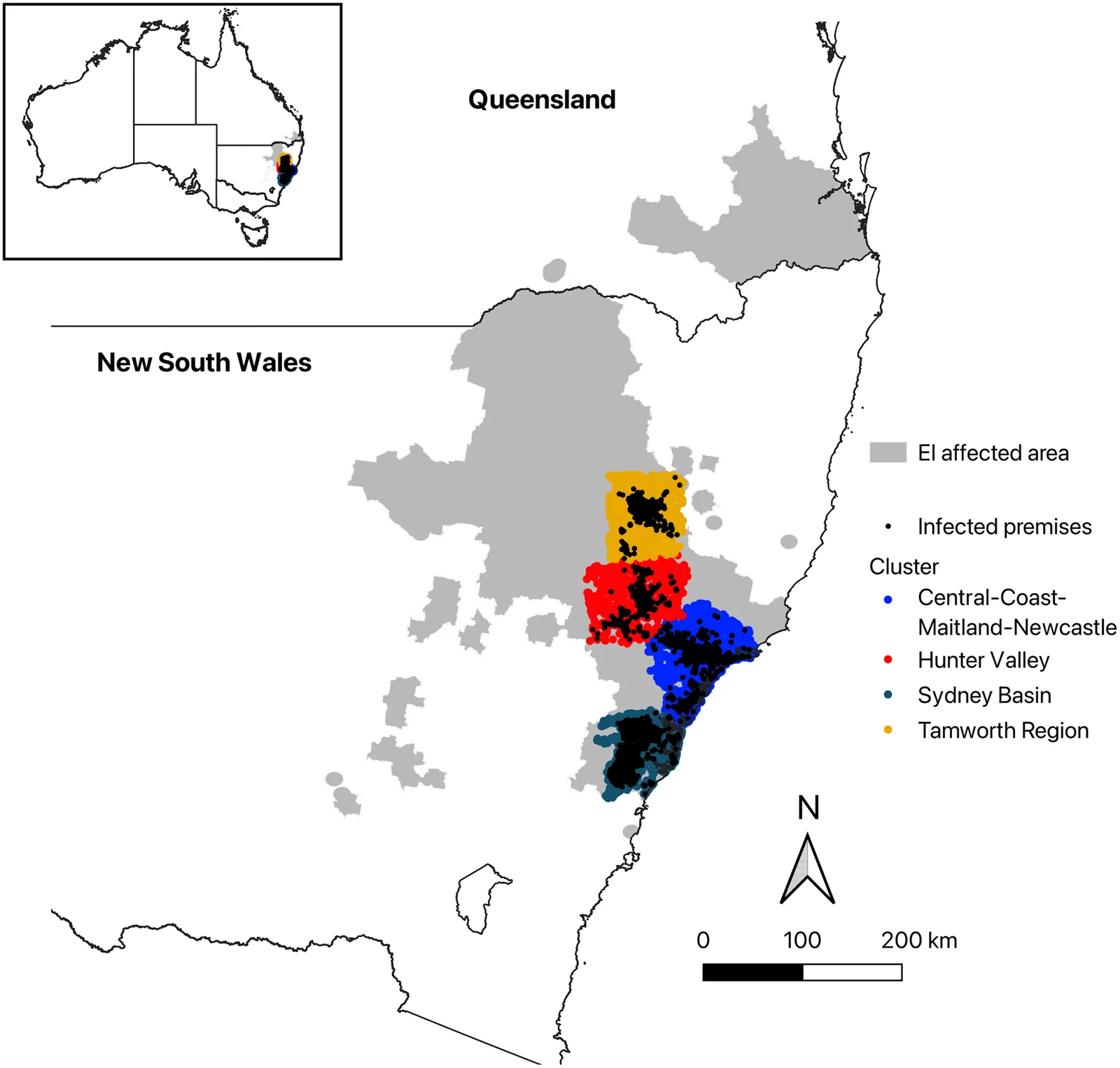
Four highly affected regions (or clusters) in the Australian 2007 equine influenza outbreak, used as study sites in the development and validation of a real-time model of equine influenza in Australia. Base map boundaries were obtained from the Australian Bureau of Statistics (ABS) Australian Statistical Geography Standard (ASGS; Edition 3, 2021–2026) digital boundary files, provided under the Creative Commons Attribution 4.0 International (CC BY 4.0) license: https://www.abs.gov.au/statistics/standards/australian-statistical-geography-standard-asgs/edition-3-july-2021-june-2026/access-and-downloads/digital-boundary-files/STE_2021_AUST_SHP_GDA2020.zip.

The Sydney, Central Coast-Maitland-Newcastle, Tamworth and Hunter Valley regions were the four most severely affected clusters in the country, covering 89% of all infectious premises (Fig 2). These clusters were as described in [35] with precise delineation implemented in the Geographic Information System QGIS QGIS Development [36] following natural geographic boundaries and purposefully ensuring separation of neighboring clusters. Descriptive statistics for each cluster are listed in (Table 1).

#### 2.4.2 Specific modelling details.

*Transmission model:* We define the component functions of Eq. 1 to capture the dynamics of the 2007 Australian Equine Influenza outbreak. First, we specify the term ω(t) in the susceptibility component (Eq. 3), to represent the time-varying reduction in premises susceptibility due to vaccination, reflecting the vaccination strategies implemented during response, where all horses on a premises were vaccinated at the same time [37]. Dates of vaccination were available for affected premises. Specifically, $\omega (t)=\text{1}-{\hat{\Omega }}_{j}(t)$, where immunity ${\hat{\Omega }}_{j}(t)\;$increases linearly from the vaccination date $t_{v,j}$ to full effectiveness Ω over 14 days, giving:


$$\begin{array}{c} {\hat{\Omega }}_{j}(t)\left\{ \begin{array}{cc} t\leq t_{v,j}, & {\hat{\Omega }}_{j}(t)=\text{0}\\ t_{v,j}<t<\left(t_{v,j}+\text{1}\text{4}\right), & {\hat{\Omega }}_{j}(t)=\Omega \left(\frac{t-t_{v,j}}{\text{1}\text{4}}\right)\\ t\geq \left(t_{v,j}+\text{1}\text{4}\right), & {\hat{\Omega }}_{j}(t)=\Omega \end{array}\right.\;. \end{array}$$

Next, we used a simple spatial transmission kernel based on Euclidean distance, to reflect the range of plausible transmission pathways during the outbreak. This formulation captures the pronounced local spread observed during the EI outbreak, including direct animal contact, fomite and windborne transmission, and potential illegal movement [38], while allowing for a small possibility of long-range transmission (e.g., via illegal movement). To account for this, we adopted a heavy-tailed Cauchy-type transmission kernel:


$$\begin{array}{c} K(j,k)=\frac{\psi }{\rho_{jk}^{\text{2}}+\psi^{\text{2}}} \end{array}$$

where $\rho_{jk}^{\text{2}}$ is the squared Euclidean distance between the centroids of premises k and j, and ψ is an unknown scaling parameter.

For each region and timepoint, our model was fitted to the EI dataset and the ABC-SMC algorithm was used to infer the posterior probability distribution for seven unknown parameters (α, $\beta_{\text{0}}$, ψ, ξ, ζ, θ, $\beta_{\text{intra}}$). Naive priors were placed on all unknown parameters.

The within-premises latent period $\text{1}/\sigma_{\text{intra}}$ was fixed at 1-day, and the recovery rate $\text{1}/\gamma_{\text{intra}}$ was fixed to 6-days based on experimental evidence [39,40]. Though, note that these can be treated as unknown parameters to be inferred by the model.

We derived exposure time $t_{e,i}$ from the assumption that the incubation period for each premises (i.e., the time between exposure and clinical onset; $t_{c,i}-t_{e,i}$) followed a Gamma distribution with a fixed shape parameter α=4 and a rate parameter λ=2, giving $\left(t_{c,i}-t_{e,i}\right)\sim \text{Gamma}(\text{4},\text{2})$. These parameters were selected to return a distribution that reflects the typical 1–3 day incubation period of equine influenza [41], with a small probability of longer incubation periods (4–6 days) as observed in ponies experimentally infectious with low doses of virus [39].

Finally, we fixed the reporting delay parameter $t_{\text{lag}}$, which specifies the number of days before the current timepoint to fit the model up to, at 3 days.

*ABC-SMC:* The number of desired particles was set as $N_{px}=\text{1}\text{0}\text{0}\text{0}$ and the number of generations to G=8 in all runs. For the largest cluster (Sydney Basin), the temporal tolerance value was set to allow an average distance of 50 cases per day for the first generation ($\epsilon_{\text{temp},\text{1}}=\text{5}\text{0}\cdot \left(T-t_{\text{detect}}+\text{1}\right)$), and the value for each subsequent generation was progressively reduced, with the tolerance for the last generation set to six cases per day ($\epsilon_{\text{temp},\text{8}}=\text{6}\cdot \left(T-t_{\text{detect}}+\text{1}\right)$). These values were scaled based on the number of premises in the cluster relative to Sydney Basin. The spatial tolerance schedule was set to allow a minimum correlation coefficient of 0.3 in the first generation ($\epsilon_{\text{spat},\text{1}}=\text{0}.\text{3}$) and progressively increasing to 0.725 in the last generation ($\epsilon_{\text{spat},\text{1}}=\text{0}.\text{7}\text{2}\text{5}$).

Tolerance schedules were set based on pilot runs to balance the tightness of fit with efficiency of sampling model parameters, noting that computational runtime can varies with data volume (i.e., population size, outbreak size), parameter dimensionality, and the tightness of tolerance thresholds. Analyses exceeding a run-time of two days (including computing cluster job queues) were terminated, as later generations can become increasingly difficult to complete when tolerances tighten and longer runs yield diminishing returns for approximating the posterior distribution (see Fig A1 in S1 File). The particles from the latest completed generation (with $N_{px}\geq \text{4}\text{0}\text{0}$) formed our model output.

All parameter values and priors used for the transmission model and the ABC-SMC algorithm can be found in Table A in S1 File.

## 3. Results

**Fig 3:**
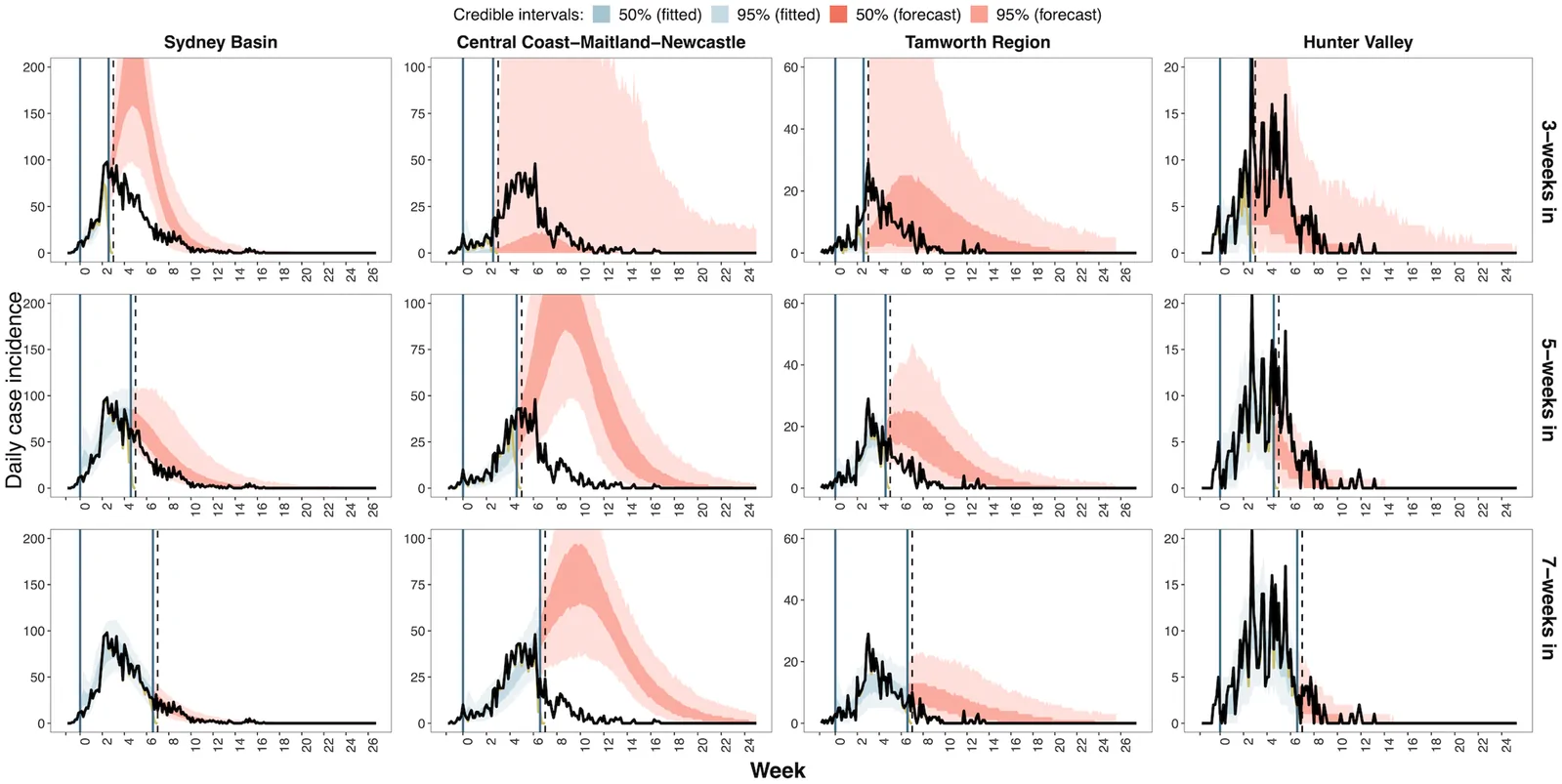
Forecast of daily EI case incidence for four outbreak clusters in Australia, shown separately for each time point (right-hand axis labels). The blue shaded regions represent model fit to the data available at the time of forecasting (dark yellow lines). The red shaded regions represent the forecast. Vertical black dashed lines indicate the time point. Vertical blue lines indicate the time window of data the model was fit to (accounting for delay with a 3-day lag). Black lines represent all reported daily case counts (i.e., all observations by end of outbreak). Shaded areas indicate the 50% and 95% percentile of 1000 simulated trajectories (of accepted particles).

We fitted our model to the four clusters of the 2007 Equine Influenza outbreak in Australia, using data from three timepoints of the outbreak (at 3, 5 and 7 weeks after first case detection) to gain an understanding of forecast performance under different amounts of data. These timepoints were chosen to reflect the observed timing of epidemic growth and peak formation, which occurred early in three out of four regions (2–5 weeks in; Fig 3).

For each fit, we generated six forecast targets: (i) future daily case counts, (ii) final size of outbreak, (iii) peak timing and size, (iv) duration of outbreak, (v) spatial pattern of infection risk, and (vi) future grid-cell case counts. The first four targets address key decision‑maker questions about overall outbreak severity and temporal trends (Section 3.1), while the latter two focus on identifying areas of immediate concern (Section 3.2). Forecast quality across different timepoints was then evaluated by quantifying how well each forecast estimated future daily case counts up to 4-weeks ahead and grid-cell case counts 1–2 week ahead (Section 3.3).

Posterior distributions of model parameters can be found in Figs B6–B9 in S1 File.

### 3.1 Outbreak severity and trend

Uncertainty in forecasts of future case trajectories (Fig 3) and associated summary statistics (Table B1 in S1 File) narrowed progressively across timepoints. While forecast uncertainty was highest at the first timepoint (3 weeks), the 95% credible intervals captured observed trends and statistics in three of the four clusters (all except Sydney).

Forecast accuracy generally improved from the second timepoint onward, with bias between observations and predictions narrowing in the Sydney, Tamworth, and Hunter Valley clusters – coinciding with the emergence of the outbreak peak in these clusters.

Despite this overall trend, the model overpredicted future case trajectories in six of the 12 forecasting scenarios, most commonly through overestimation of peak timing (five of six cases). These overpredictions occurred under two main conditions: (i) when the outbreak peak had not yet formed (Sydney at 3 weeks; CCMN at 5 and 7 weeks), and (ii) when the peak had formed but was poorly captured by the model fit (Sydney 5 weeks; Tamworth at 5 and 7 weeks). The magnitude of overestimation varied from marginal when post-peak fits where poor, where observed data lay near the lower bound of the 95% credible interval, to substantial in pre-peak scenarios, with observations falling well below the credible interval. By contrast, forecasts for Sydney and Hunter Valley at the final timepoint (7 weeks) were both more accurate and more precise, reflecting improved model fit to the outbreak peak.

### 3.2 Areas of immediate concern

**Fig 4:**
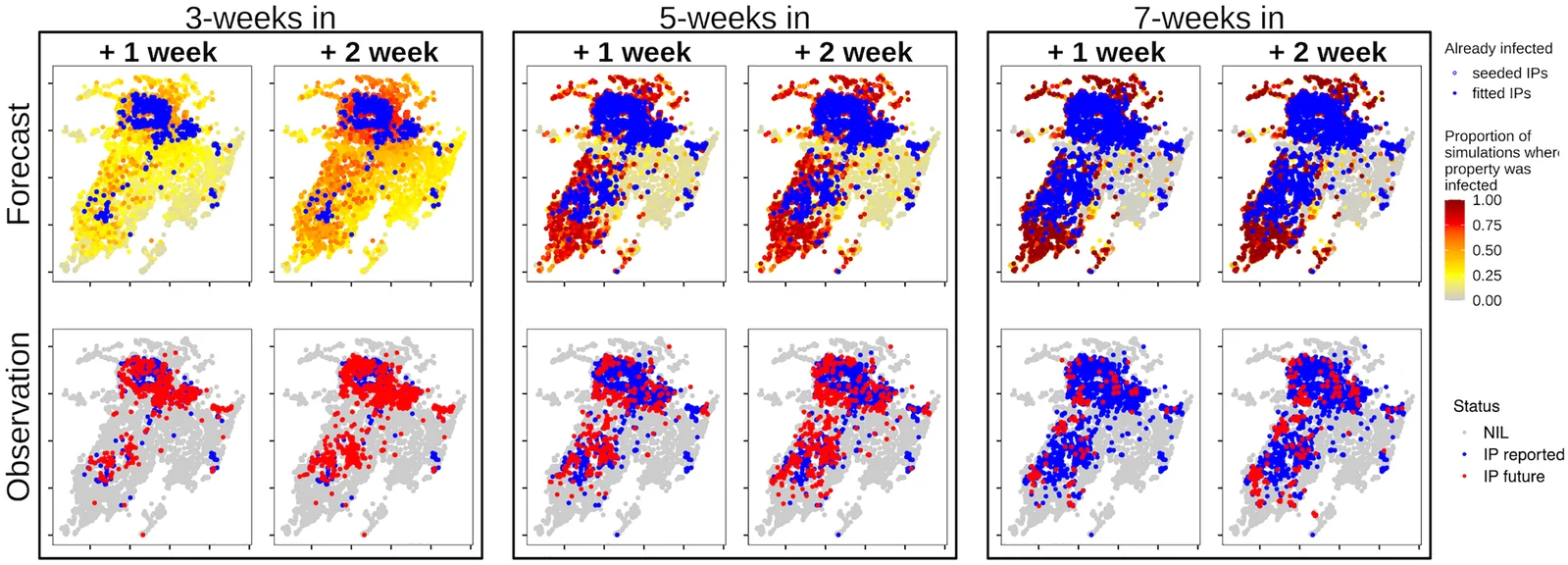
Comparison of simulated and observed outbreaks for the Sydney Basin region for each timepoint. Spatial maps showing proportion of runs (out of 1000 simulation runs) in which a premises was infected between the date of first detection and 1-week and 2-weeks ahead from the timepoint (top panel); compared to the single observed realisation of the outbreak at the same respective dates (bottom panel; “NIL”: non-infectious premises; “IP reported”: infectious premises reported before the timepoint; “IP future”: infectious premises reported after the timepoint). Plots for other regions and timepoints can be found in Figs B3–B5 in S1 File.

To address the question “Which premises are likely to be affected?”, we consider the risk that a given premises becomes infected during the current outbreak. This was estimated by the proportion of simulations (out of 1000) in which a presently susceptible premises was infected (i.e., transition to the exposed state) between the date of first detection and (i) 1-week ahead from the observation date (i.e., $t_{\text{today}}$), and (ii) 2-weeks from the observation date. These are shown as spatial risk maps in Fig 4 and Figs B3–B5 in S1 File, demonstrating how the risk estimate progresses with time within each cluster, and how they compare to the observed outbreak.

Overall, spatial forecasts across clusters and timepoints generally provided clear indication of high-risk areas in the next 1–2 weeks, with the exception of CCMN forecasts at 3 weeks, which were too imprecise to meaningfully identify risk areas. Spatial risk predictions of 1- and 2-week ahead infection risk captured broad patterns of future transmissions in terms of relative risk, but were less informative for predicting exact locations of new cases. In Sydney, forecasts indicated a more widespread outbreak, with predicted future cases occurring further from already reported cases (Fig 4).

### 3.3 Forecast performance

#### 3.3.1 Four-weeks ahead daily case counts.

**Fig 5:**
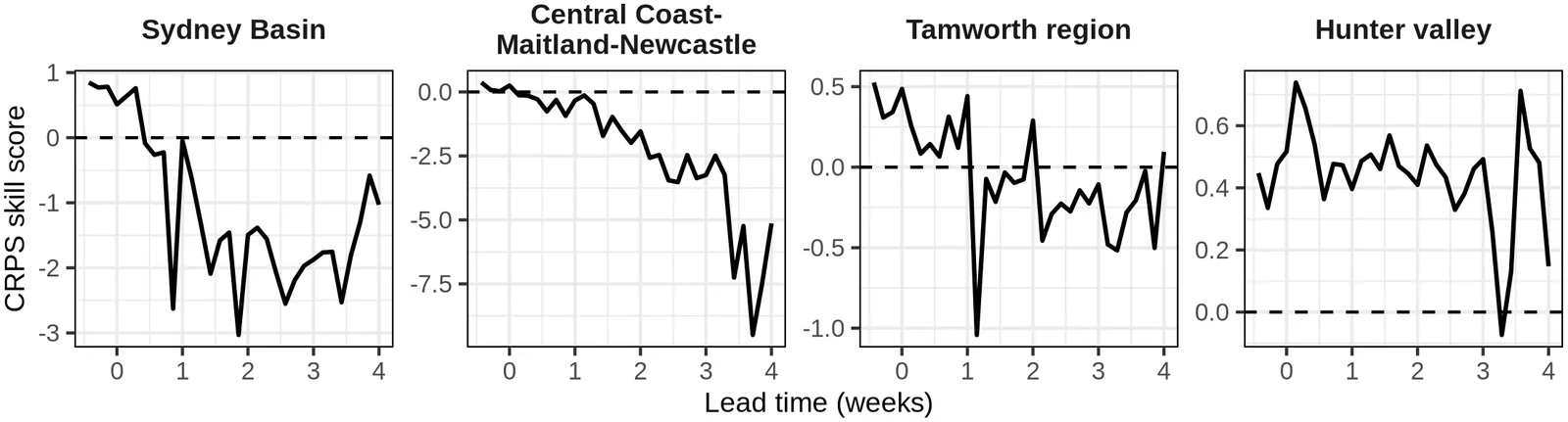
Forecast skill of EI daily case counts by lead time for four outbreak clusters in Australia, averaged across all three timepoints. Positive skill scores indicate that the forecasts outperformed the naive benchmark, with a maximum possible score of one (i.e., perfect accuracy and no uncertainty). Negative skill scores indicate underperformance relative to the naive benchmark, with a minimum score of −∞. Note that the y‑axis scale differs between subplots, and the horizontal dashed line denotes zero skill.

Overall, forecast skill was higher for shorter lead times (i.e., forecasts closer to the present) across all four outbreak clusters (Figs 5 and B10 in S1 File). On average across timepoints, forecasts outperformed the naive benchmark (i.e., the most recently observed case count – 3-days before the day of prediction) for 0.5 and 1-week lead times in the Sydney and Tamworth clusters, respectively. In the smallest cluster, Hunter Valley, forecasts outperformed the naive benchmark for almost all lead times, which reported a maximum of 17 cases a day. The naive benchmark outperformed the forecasts for lead times beyond 0 weeks in CCMN, where the most recently observed case count was a better predictor of the future case counts.

For longer lead times, the most recently observed case count – which under-reported cases ultimately recorded for the day (even with a 3-day lag; see Fig 3) – better aligned with future observations showing a decrease in daily cases, and increased their skill relative to the model’s forecasts. Forecast skill across all clusters was highest when daily case counts were 0 or ≥30, and was lower for days where 1–9 cases were reported (Fig B11 in S1 File).

#### 3.3.2 One to two weeks ahead grid-cell case counts.

Spatial forecast skill was evaluated separately for grid cells with at least one reported case versus those with none. Non-zero cells represent the current outbreak extent (*active zone*), while zero-case cells represent locations not yet affected (*inactive zone*). Skill scores have different interpretations across zones. In the active zone, positive skill indicates improvement over the naïve benchmark. In the inactive zone, performance better than or equal to (skill ≥ 0) the naïve benchmark is preferred, noting the naïve benchmark cannot be outperformed where no new cases are observed as it trivially achieves perfect CRPS by always propagating zeroes.

**Fig 6:**
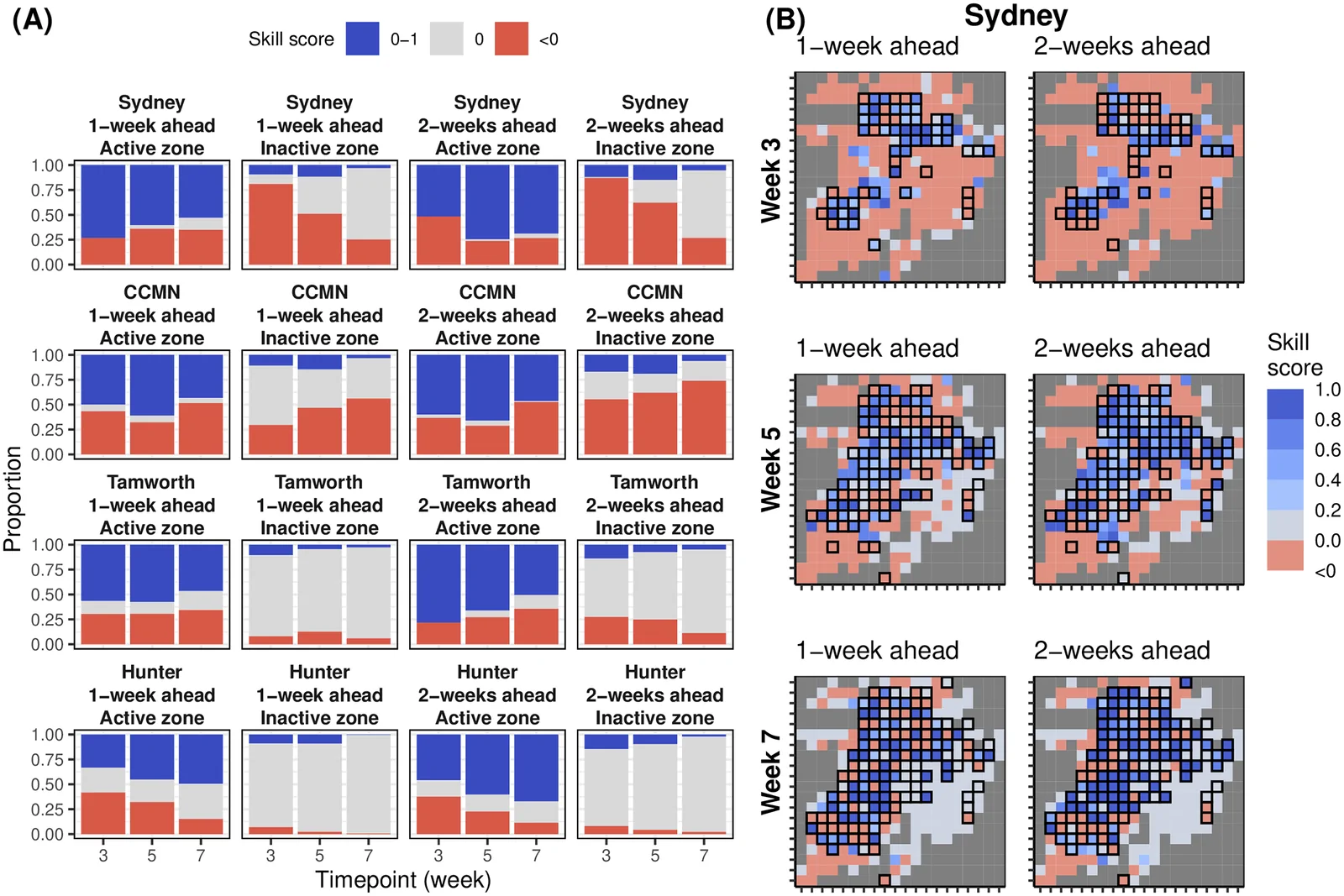
Spatial forecast skill for 1- and 2-week ahead EI case counts. (A) Summary of proportion of grids where the forecast outperformed (CRPS skill score of >0), performed the same (=0), or underperformed (<0) the naive benchmark within the *active* outbreak zone (i.e., observed case counts >0) versus the *inactive* zone (cases = 0). (B) Spatial distribution of forecast skill for 1- and 2-week ahead case counts for the Sydney Basin; black outlines denote grids within the active zone. See Fig B12 in S1 File for other clusters.

Within the *active* zone, the model achieved positive skill (skill > 0) in a greater proportion of grid cells than those where performance is equal to or worse than the naïve benchmark (skill ≤ 0) in most scenarios (Fig 6A). Two-week-ahead forecasts showed marginally better performance than one-week-ahead forecasts, although no trend was evident across timepoints.

Performance in the *inactive* zone varied substantially by cluster size. The model performed as well as or better than the naïve benchmark (skill ≥ 0) in 19–75% of grid cells for Sydney and 44–70% for CCMN in one-week-ahead forecasts across timepoints (Fig 6 and Fig B12 in S1 File). The model performed considerably better in the two smaller clusters (Tamworth and Hunter), with non-negative skill in more than 90% of grid cells for one-week-ahead forecasts across timepoints. Performance improved over timepoints in all clusters except CCMN, where skill deteriorated, mirroring patterns observed in daily case count forecasts (Fig 3). Across all clusters and timepoints, one-week-ahead forecasts outperformed two-week-ahead forecasts in the inactive zone.

## 4. Discussion

In this work, we introduced a real-time forecasting framework that can be generalised to any infectious disease of livestock. Application to premises-level data from the 2007 EI outbreak in Australia demonstrated its potential for providing timely and informative spatiotemporal forecasts for different amounts of data and in different regions/clusters. We use findings from our case study to identify potential directions for further investigation, with a view to continuously improve forecast performance and extend its application to other animal diseases in future work.

### 4.1 Principal findings

Our initial evaluation shows that forecast performance generally improved with time as more data became available. Forecast *uncertainty* reduced with time as case data increased in volume, which enabled more precise estimation in parameters describing disease spread (see Table B2 and Figs B6–B9 in S1 File). Early in the outbreak (3-weeks), uncertainty tended to be high when reported caseloads up to the time of forecasting was low, as seen in the three smaller clusters where daily case incidence was below 10 at the first timepoint (Figs 3 and 4, and Figs B3–B5 in S1 File). Prediction uncertainty is a common problem early in the course of an epidemic [42,43], when intervention decisions are most consequential. However, more data, particularly after the peak of the epidemic, results in better model fits and characterisation of the wave, a pattern demonstrated across epidemiological models (e.g., [6,44]). Incorporating expert knowledge into parameter prior distributions from the outset can improve forecast precision under data scarcity [15]. Forecast *accuracy* improved after outbreak peak formation, as the impact of control measures became evident in the case data. In particular, the early movement ban enacted once the first case was detected substantially reduced background spread [16]. Hence, forecasts made just before peak formation (during the exponential growth phase) that overpredicted future case counts substantially (i.e., Sydney Basin at 3-weeks, CCMN at 5- and 7-weeks), plausibly reflects what could have happened in the absence of movement restrictions and other controls. In scenarios where the peak had formed but was poorly captured by the model fit (Sydney at 5-weeks; Tamworth at 5- and 7-weeks), case counts were also overpredicted but to a smaller degree, indicating shifts in underlying dynamics that were not explicitly modelled. These findings align with the well documented tendency of epidemic models to overpredict case numbers due to unidentified population heterogeneity (e.g., [45,46]). Accounting for changing control policies in the model (either explicitly or via time-varying background transmission rates; e.g., [47]) or restricting model fitting to the period following the most recent policy change is an important direction for future work, but requires timely and jurisdiction-specific policy data.

Forecasts of future daily case counts and spatial case counts (in the inactive zone) exhibited highest skill at shorter lead times, supporting greater confidence in nearer-term projections. This agrees with the general expectation that forecasts performance degrades as they predict further ahead [48], as shown in both theoretical work (e.g., [42]) and empirical studies across outbreaks [49,50]. Thus, decisions may be better informed by nearer-term predictions (1–2 weeks ahead) than by summary measures of overall outbreak dynamics (e.g., final size, duration, peak timing). That said, forecasts of daily case counts often underperformed the naïve benchmark, reflecting opposing biases: the naïve model tends to underestimate future cases due to reporting delays, while the mechanistic model more often overestimates it. Thus, over longer time horizons (i.e., 4-weeks ahead), particularly during the outbreak’s deceleration phase (when daily cases fall significantly), the naive benchmark can achieve higher accuracy. Indeed, the naïve model represents the simplest baseline in time‑series forecasting and is not trivial to beat (e.g., [51]). As a risk‑evaluation tool, however, the mechanistic model provides a substantive advantage by representing the potential for large outbreaks (e.g., ≥ 30 daily cases) and estimating peak timing and size, thereby offering a meaningful baseline for continued model improvement.

Despite these general trends, performance varied substantially between regional clusters. Plausible sources of variation include regional differences in premises-level horse density, timing and intensity of movement control policies, biosecurity and other policy compliance, and population data accuracy [34,38,52]. Further investigation is needed to disentangle the individual contributions of these factors to forecast performance. Variation in forecast performance across time and locations is a known feature of single models, as previously demonstrated for forecasting COVID-19 [51], influenza [53], Ebola [49] and dengue fever outbreaks [54]. The same studies show that ensemble approaches combining forecasts from multiple models often (if not always) improve forecast accuracy and stability, and our model could contribute to such frameworks.

### 4.2 Study strengths

A key strength of our modelling framework is the ability to provide reasonable spatial forecasts throughout the outbreak. While individual-premises infection risk predictions were noisy, the model nonetheless captured broad spatial patterns of relative risk, showed net improvements over the naïve benchmark for predicting case counts in the active outbreak zone in most scenarios, and successfully predicted realised growth in outbreak extent in the smaller clusters (Tamworth and Hunter Valley). The main exceptions were Sydney and CCMN, where infection risk was predicted but not realised, likely due to effective control measures that were not explicitly modelled and is exacerbated by the skill metric penalising any non-zero risk relative to a zero-persistence benchmark. Importantly, the framework’s value lies in indicating *where* relative risk of future cases is higher or lower, rather than in precise prediction of case numbers, to guide geographically targeted control and surveillance. Overall, spatial forecast performance was notable given the use of a rather crude ABC-SMC fitting algorithm, uncertainty in spatial kernel parameter estimates, and (in some scenarios) overprediction in daily case counts. We expect spatial forecast skill to improve with better model representation of intervention effects on future transmission dynamics.

Another strength is the use of an ABC-SMC fitting approach, which offers greater generalisability across outbreak contexts and improved computational efficiency relative to full MCMC methods. The simple distance metrics used to fit daily and grid-cell case counts proved effective and readily transferable to any model adaptation. Importantly, forecasts were generated in near-real-time: for the largest region (7387 premises) stabilised parameter estimates were obtained within approximately one day (typically generations 4–6) on a 50-node computing cluster. Having established forecasting capability a clear next step is to further improve computational efficiency through more advanced or tailored fitting algorithms. These features contrast with full MCMC approaches that have been used in similar modelling frameworks (e.g., [15,16]), which can be much slower to program and perform due to repeated likelihood calculations [55].

### 4.3 Limitations and challenges

In our initial attempt at configuring the framework for our EI case study, we prioritised model parsimony to suit a rapid model fitting process and to suit the data available. As such, there are several factors that we have not considered here. First, we assumed that background/baseline spread rates do not change over time. Time-dependent background or baseline spread rates are often used to account for the impact of future interventions and lifting of restrictions (e.g., [28,47]). However, inference of time-varying rates as unknown parameters requires assumptions on future spread rates or between-premises movement data to inform these rates (e.g., [15]), which we did not have for the EI outbreak. Secondly, there may some level of misspecification bias in our model formulation of factors driving disease spread as parameter sensitivity varied a great deal (see Figs B6–B9 in S1 File). Alternative model formulations should be explored in future work and could be accompanied by sensitivity analyses as a guide. Our study serves as a benchmark from which alternative formulations can be evaluated against using the skill scoring approach we have developed. Third, we corrected for delays in case reporting by starting the forecast three days prior to the timepoint (i.e., $t_{\text{lag}}=\text{3}$), which appeared to be sufficient in mitigating the effect of reporting delays on forecast performance in our case. However, this simple correction for delays will likely not apply in many situations, where often, delays between symptom onset and case reporting are context specific and are modelled based on available real-time data (e.g., [28]). Accounting for partial observations (e.g., right-censoring) can readily be done in our modelling framework and should be a priority in future work.

Apart from modelling decisions that may have limited model performance, we also noticed issues in our underlying data. We found location information for a number of horse premises to be inaccurate, which is a common problem across animal health surveillance data [56]. This would have affected estimation of transmission distance and horse density (as premises area was estimated from locations, [16], and consequently, parameter estimation and forecast reliability. Thus, regular updates and verification of livestock industry databases is critical, which can be assisted by emerging machine learning approaches [56,57].

### 4.4 Implications and future work

We have introduced a modelling framework designed to provide real-time forecast during emerging animal health crises and to be rapidly adaptable to any outbreak context. Our results highlight several considerations when using our model to guide decisions. First, *greater weight should be placed on short-term forecasts of up to two weeks ahead*, while longer-term forecasts and summary metrics (e.g., peak size and final size) should be interpreted as indicative scenarios rather than precise projections, particularly for early timepoints. Second, *decisions should consider whether current or upcoming policy changes are represented in the model*; if not, outputs should be interpreted as counterfactual or lagging representations of reality. Third, *prioritise predictions of broad spatial risk patterns to support geographically targeted surveillance and control*, rather than precise forecasts of which premises will be infected or exact case counts, which are inherently more uncertain. Finally, *the ability to generate reliable and timely predictions ultimately hinges on data availability and quality*. Beyond population data inaccuracies, common issues such as reporting delays (e.g., [28,58]) and unreported or undetected cases (e.g., [18]) are well known to diminish forecast performance.

The modelling framework and initial findings presented here opens doors for follow-up activities, which should contribute to improving animal disease forecasting capabilities and building preparedness in model deployment during future crises. Extending the framework to incorporate airborne- and vector-mediated spread (e.g., [46]) will allow application to animal disease such as FMD, LSD and HPAI, which are of high concern. Use of our framework for real-time evaluation of control strategies to guide choice of intervention can be explored (e.g., [18]), noting that it already possesses the capacity to do so for vaccination and culling measures (and can be easily extended to other intervention types). In terms of improving forecast performance, numerous refinements to the epidemic model and ABC-SMC fitting algorithm can be explored, including: the addition of an observation model to account for incomplete data (e.g., undetected infections, reporting delays), integration of movement/contact network data to predict new seeding events, a spatiotemporal distance measure that compares the observed and simulated spatial distribution of infections at multiple time intervals (e.g., [25]). In addition, investigating the importance of data accuracy and completeness to forecast reliability is also a critical area of further research. Finally, as recognised in public health, there is a similar need for ongoing collaborations between modellers and animal health practitioners to fully leverage modelling tools to support outbreak response. Simulation (or synthetic) exercises held in collaboration with practitioners can provide valuable experience in real-time deployment of our modelling framework, adaptation to unexpected situations, working with incomplete and evolving data, and effective communication of forecast outputs to decision-makers. Several activities outlined here are underway through ongoing partnerships with Australian government and industry institutions, aimed at strengthening the country’s capacity for rapid decision-support during animal disease outbreaks.

## 5. Data and code accessibility

The C++ code implementing the simulation and R code implementing the analysis used in this study may be found at https://gitlab.unimelb.edu.au/mtheng/livestock-forecasting, released under the GNU Public License v. 3. Access to the underlying data is restricted to preserve the confidentiality of premises locations and owing to confidentiality requirements and legal agreements with the NSW Department of Primary Industries. An example subset with randomised premises locations is provided in the repository to demonstrate the workflow. Requests for these data should be directed to the NSW Department of Primary Industries (animal.biosecurity@dpird.nsw.gov.au).

## Supporting information

S1 File Supplementary material. (PDF)

## References

[1] Haydon DT, Kao RR, Kitching RP. The UK foot-and-mouth disease outbreak - the aftermath. Nat Rev Microbiol. 2004;2(8):675–81. doi: 10.1038/nrmicro960 15263902

[2] Wille M, Barr IG. Resurgence of avian influenza virus. Science. 2022;376(6592):459–60. doi: 10.1126/science.abo1232 35471045

[3] Roest HIJ, Tilburg JJHC, van der Hoek W, Vellema P, van Zijderveld FG, Klaassen CHW, et al. The Q fever epidemic in The Netherlands: history, onset, response and reflection. Epidemiol Infect. 2011;139(1):1–12. doi: 10.1017/S0950268810002268 20920383

[4] Azeem S, Sharma B, Shabir S, Akbar H, Venter E. Lumpy skin disease is expanding its geographic range: a challenge for Asian livestock management and food security. Veterinary J. 2022;279:105785. 10.1016/j.tvjl.2021.105785 34915159

[5] Pell B, Kuang Y, Viboud C, Chowell G. Using phenomenological models for forecasting the 2015 Ebola challenge. Epidemics. 2018;22:62–70. doi: 10.1016/j.epidem.2016.11.002 27913131

[6] Shanafelt DW, Jones G, Lima M, Perrings C, Chowell G. Forecasting the 2001 foot-and-mouth disease epidemic in the UK. Ecohealth. 2018;15(2):338–47. doi: 10.1007/s10393-017-1293-2 29238900 PMC6132414

[7] Chowell G, Fenimore PW, Castillo-Garsow MA, Castillo-Chavez C. SARS outbreaks in Ontario, Hong Kong and Singapore: the role of diagnosis and isolation as a control mechanism. J Theor Biol. 2003;224(1):1–8. doi: 10.1016/s0022-5193(03)00228-5 12900200 PMC7134599

[8] Chowell G, Castillo-Chavez C, Fenimore PW, Kribs-Zaleta CM, Arriola L, Hyman JM. Model parameters and outbreak control for SARS. Emerg Infect Dis. 2004;10(7):1258–63. doi: 10.3201/eid1007.030647 15324546 PMC3323341

[9] Bradhurst RA, Roche SE, East IJ, Kwan P, Garner MG. A hybrid modeling approach to simulating foot-and-mouth disease outbreaks in Australian livestock. Front Environ Sci. 2015;3. doi: 10.3389/fenvs.2015.00017

[10] Lessler J, Azman AS, Grabowski MK, Salje H, Rodriguez-Barraquer I. Trends in the mechanistic and dynamic modeling of infectious diseases. Curr Epidemiol Rep. 2016;3(3):212–22. doi: 10.1007/s40471-016-0078-4 32226711 PMC7100697

[11] Lutz CS, Huynh MP, Schroeder M, Anyatonwu S, Dahlgren FS, Danyluk G, et al. Applying infectious disease forecasting to public health: a path forward using influenza forecasting examples. BMC Public Health. 2019;19(1):1659. doi: 10.1186/s12889-019-7966-8 31823751 PMC6902553

[12] Arenas A, Cota W, Gómez-Gardeñes J, Gómez S, Granell C, Matamalas JT, et al. Modeling the spatiotemporal epidemic spreading of COVID-19 and the impact of mobility and social distancing interventions. Phys Rev X. 2020;10(4). doi: 10.1103/physrevx.10.041055

[13] Chang SL, Harding N, Zachreson C, Cliff OM, Prokopenko M. Modelling transmission and control of the COVID-19 pandemic in Australia. Nat Commun. 2020;11(1):5710. doi: 10.1038/s41467-020-19393-6 33177507 PMC7659014

[14] Stevenson MA, Sanson RL, Stern MW, O’Leary BD, Sujau M, Moles-Benfell N, et al. InterSpread Plus: a spatial and stochastic simulation model of disease in animal populations. Preventative Veterinary Medicine. 2013;109(1–2):10–24. 10.1016/j.prevetmed.2012.08.015 22995473

[15] Jewell CP, Kypraios T, Neal P, Roberts GO. Bayesian analysis for emerging infectious diseases. Bayesian Anal. 2009;4(3). doi: 10.1214/09-ba417

[16] Firestone SM. Epidemiological investigations into the 2007 outbreak of equine influenza in Australia. The University of Sydney; 2012.

[17] Minh PQ, Stevenson MA, Jewell C, French N, Schauer B. Spatio-temporal analyses of highly pathogenic avian influenza H5N1 outbreaks in the Mekong River Delta, Vietnam, 2009. Spat Spatiotemporal Epidemiol. 2011;2(1):49–57. doi: 10.1016/j.sste.2010.11.001 22749548

[18] Probert WJM, Jewell CP, Werkman M, Fonnesbeck CJ, Goto Y, Runge MC, et al. Real-time decision-making during emergency disease outbreaks. PLoS Comput Biol. 2018;14(7):e1006202. doi: 10.1371/journal.pcbi.1006202 30040815 PMC6075790

[19] Drovandi CC, Pettitt AN, McCutchan RA. Exact and approximate bayesian inference for low integer-valued time series models with intractable likelihoods. Bayesian Anal. 2016;11(2). doi: 10.1214/15-ba950 26584211

[20] Gillespie DT. Exact stochastic simulation of coupled chemical reactions. J Phys Chem. 1977;81(25):2340–61. doi: 10.1021/j100540a008

[21] Rorres C, Pelletier STK, Keeling MJ, Smith G. Estimating the kernel parameters of premises-based stochastic models of farmed animal infectious disease epidemics using limited, incomplete, or ongoing data. Theor Popul Biol. 2010;78(1):46–53. doi: 10.1016/j.tpb.2010.04.003 20452368 PMC2902694

[22] Boender GJ, Hagenaars TJ. Common features in spatial livestock disease transmission parameters. Sci Rep. 2023;13(1):3550. doi: 10.1038/s41598-023-30230-w 36864168 PMC9981765

[23] Golightly A, Wadkin LE, Whitaker SA, Baggaley AW, Parker NG, Kypraios T. Accelerating Bayesian inference for stochastic epidemic models using incidence data. Stat Comput. 2023;33(6). doi: 10.1007/s11222-023-10311-6

[24] McKinley TJ, Vernon I, Andrianakis I, McCreesh N, Oakley JE, Nsubuga RN, et al. Approximate Bayesian computation and simulation-based inference for complex stochastic epidemic models. Statist Sci. 2018;33(1). doi: 10.1214/17-sts618

[25] Minter A, Retkute R. Approximate bayesian computation for infectious disease modelling. Epidemics. 2019;29:100368. 31563466 10.1016/j.epidem.2019.100368

[26] Beaumont MA. Approximate bayesian computation in evolution and ecology. Annu Rev Ecol Evol Syst. 2010;41(1):379–406. doi: 10.1146/annurev-ecolsys-102209-144621

[27] Gneiting T, Katzfuss M. Probabilistic forecasting. Ann Rev Stat Appl. 2014;1(1):125–51. doi: 10.1146/annurev-statistics-062713-085831

[28] Moss R, Price DJ, Golding N, Dawson P, McVernon J, Hyndman RJ, et al. Forecasting COVID-19 activity in Australia to support pandemic response: May to October 2020. Sci Rep. 2023;13(1):8763. doi: 10.1038/s41598-023-35668-6 37253758 PMC10228456

[29] Moloney BJ. Overview of the epidemiology of equine influenza in the Australian outbreak. Aust Vet J. 2011;89 Suppl 1:50–6. doi: 10.1111/j.1751-0813.2011.00748.x 21711290

[30] Callinan I. Equine influenza-the August 2007 outbreak in Australia-report of the equine influenza inquiry. 2008.

[31] Glanville RJ, Christie B. High-level coordination and strategy in the 2007 equine influenza outbreak response. Aust Vet J. 2011;89 Suppl 1:97–100. doi: 10.1111/j.1751-0813.2011.00759.x 21711302

[32] Hafi A, Gomboso J, Hean R, Scott F, Arthur T, Rahman N. Estimating the value of Australian biosecurity arrangements for equine influenza since the 2007 outbreak. Canberra: ABARES; 2020.

[33] Garner MG, Cowled B, East IJ, Moloney BJ, Kung NY. Evaluating the effectiveness of early vaccination in the control and eradication of equine influenza--a modelling approach. Prev Vet Med. 2011;99(1):15–27. doi: 10.1016/j.prevetmed.2010.02.007 20236718

[34] Cowled B, Ward MP, Hamilton S, Garner G. The equine influenza epidemic in Australia: spatial and temporal descriptive analyses of a large propagating epidemic. Prev Vet Med. 2009;92(1–2):60–70. doi: 10.1016/j.prevetmed.2009.08.006 19748691

[35] Firestone SM, Ward MP, Christley RM, Dhand NK. The importance of location in contact networks: Describing early epidemic spread using spatial social network analysis. Prev Vet Med. 2011;102(3):185–95. doi: 10.1016/j.prevetmed.2011.07.006 21852007

[36] QGIS Development Team. QGIS geographic information system. Open Source Geospatial Foundation; 2024.

[37] Perkins NR, Webster WR, Wright T, Denney I, Links I. Vaccination program in the response to the 2007 equine influenza outbreak in Australia. Aust Vet J. 2011;89 Suppl 1:126–34. doi: 10.1111/j.1751-0813.2011.00766.x 21711310

[38] Davis J, Garner MG, East IJ. Analysis of local spread of equine influenza in the Park Ridge region of Queensland. Transbound Emerg Dis. 2009;56(1–2):31–8. doi: 10.1111/j.1865-1682.2008.01060.x 19200296

[39] Mumford JA, Hannant D, Jessett DM. Experimental infection of ponies with equine influenza (H3N8) viruses by intranasal inoculation or exposure to aerosols. Equine Vet J. 1990;22(2):93–8. doi: 10.1111/j.2042-3306.1990.tb04217.x 2156688

[40] Daly JM, MacRae S, Newton JR, Wattrang E, Elton DM. Equine influenza: a review of an unpredictable virus. Vet J. 2011;189(1):7–14. doi: 10.1016/j.tvjl.2010.06.026 20685140

[41] Paillot R, Hannant D, Kydd JH, Daly JM. Vaccination against equine influenza: quid novi?. Vaccine. 2006;24(19):4047–61. doi: 10.1016/j.vaccine.2006.02.030 16545507

[42] Castro M, Ares S, Cuesta JA, Manrubia S. The turning point and end of an expanding epidemic cannot be precisely forecast. Proc Natl Acad Sci U S A. 2020;117(42):26190–6. doi: 10.1073/pnas.2007868117 33004629 PMC7585017

[43] Wilke CO, Bergstrom CT. Predicting an epidemic trajectory is difficult. Proc Natl Acad Sci U S A. 2020;117(46):28549–51. doi: 10.1073/pnas.2020200117 33144506 PMC7682412

[44] Alberti T, Faranda D. On the uncertainty of real-time predictions of epidemic growths: a COVID-19 case study for China and Italy. Commun Nonlinear Sci Numer Simul. 2020;90:105372. doi: 10.1016/j.cnsns.2020.105372 32834701 PMC7263229

[45] Ong JBS, Chen MI-C, Cook AR, Lee HC, Lee VJ, Lin RTP, et al. Real-time epidemic monitoring and forecasting of H1N1-2009 using influenza-like illness from general practice and family doctor clinics in Singapore. PLoS One. 2010;5(4):e10036. doi: 10.1371/journal.pone.0010036 20418945 PMC2854682

[46] Jewell CP, Brown RG. Bayesian data assimilation provides rapid decision support for vector-borne diseases. J R Soc Interface. 2015;12(108):20150367. doi: 10.1098/rsif.2015.0367 26136225 PMC4528604

[47] Dehning J, Zierenberg J, Spitzner FP, Wibral M, Neto JP, Wilczek M, et al. Inferring change points in the spread of COVID-19 reveals the effectiveness of interventions. Science. 2020;369(6500):eabb9789. doi: 10.1126/science.abb9789 32414780 PMC7239331

[48] Tong H. Non-linear time series: A dynamical system approach. Clarendon Press; 1990.

[49] Viboud C, Sun K, Gaffey R, Ajelli M, Fumanelli L, Merler S, et al. The RAPIDD ebola forecasting challenge: synthesis and lessons learnt. Epidemics. 2018;22:13–21. 28958414 10.1016/j.epidem.2017.08.002 PMC5927600

[50] Scarpino SV, Petri G. On the predictability of infectious disease outbreaks. Nat Commun. 2019;10(1):898. doi: 10.1038/s41467-019-08616-0 30796206 PMC6385200

[51] Cramer EY, Ray EL, Lopez VK, Bracher J, Brennen A, Castro Rivadeneira AJ, et al. Evaluation of individual and ensemble probabilistic forecasts of COVID-19 mortality in the United States. Proc Natl Acad Sci U S A. 2022;119(15):e2113561119. doi: 10.1073/pnas.2113561119 35394862 PMC9169655

[52] Firestone SM, Schemann KA, Toribio J-ALML, Ward MP, Dhand NK. A case-control study of risk factors for equine influenza spread onto horse premises during the 2007 epidemic in Australia. Prev Vet Med. 2011;100(1):53–63. doi: 10.1016/j.prevetmed.2011.03.002 21481961

[53] Reich NG, Brooks LC, Fox SJ, Kandula S, McGowan CJ, Moore E, et al. A collaborative multiyear, multimodel assessment of seasonal influenza forecasting in the United States. Proc Natl Acad Sci U S A. 2019;116(8):3146–54. doi: 10.1073/pnas.1812594116 30647115 PMC6386665

[54] Johansson MA, Apfeldorf KM, Dobson S, Devita J, Buczak AL, Baugher B, et al. An open challenge to advance probabilistic forecasting for dengue epidemics. Proc Natl Acad Sci U S A. 2019;116(48):24268–74. doi: 10.1073/pnas.1909865116 31712420 PMC6883829

[55] McKinley T, Cook AR, Deardon R. Inference in epidemic models without likelihoods. The International Journal of Biostatistics. 2009;5(1). doi: 10.2202/1557-4679.1171

[56] Sheffield KJ, Hunnam JC, Cuzner TN, Morse-McNabb EM, Sloan SM, Nunan J, et al. Automated identification of intensive animal production locations from aerial photography. Aust Vet J. 2018;96(9):323–31. doi: 10.1111/avj.12732 30152061

[57] van Andel M, Jewell C, McKenzie J, Hollings T, Robinson A, Burgman M, et al. Predicting farm-level animal populations using environmental and socioeconomic variables. Prev Vet Med. 2017;145:121–32. doi: 10.1016/j.prevetmed.2017.07.005 28903868

[58] Reich NG, Lauer SA, Sakrejda K, Iamsirithaworn S, Hinjoy S, Suangtho P, et al. Challenges in real-time prediction of infectious disease: a case study of dengue in Thailand. PLoS Negl Trop Dis. 2016;10(6):e0004761. doi: 10.1371/journal.pntd.0004761 27304062 PMC4909288

