# Supplementary material for: A real-time forecasting framework for emerging infectious diseases affecting animal populations
Source: PLoS Comput Biol. 2026 Sep 10;22(9):e1014716. doi: 10.1371/journal.pcbi.1014716 (PMC13561369; doi:10.1371/journal.pcbi.1014716)
Supplement: Supplementary file 1 [file pcbi.1014716.s001.pdf]

A real-time forecasting framework for emerging infectious diseases affecting animal populations: Supplementary Material

Meryl Theng[1,2,3,*] Simin Lee[3] Andrew C. Breed[4,5]
Sharon Roche[5] Emily Sellens[5] Catherine Fraser[6]
Kelly Wood[6] Christopher Jewell[7] Mark A. Stevenson[3]
Chris Baker[1,2] Simon M. Firestone[3]

[1] School of Mathematics and Statistics, The University of Melbourne, Melbourne, Victoria, Australia

[2] Melbourne Centre for Data Science, The University of Melbourne, Melbourne, Victoria, Australia

[3] Melbourne Veterinary School, The University of Melbourne, Melbourne, Victoria, Australia

[4] Epidemiology, Surveillance and Laboratory Section, Australian Government Department of Agriculture, Fisheries and Forestry, Canberra, Australian Capital Territory, Australia

[5] School of Veterinary Science, University of Queensland, Brisbane, Queensland, Australia

[6] New South Wales Department of Primary Industries, Orange, New South Wales,Australia

[7] Department of Mathematics and Statistics, Lancaster University, Bailrigg, Lancaster, United Kingdom

[*] Correspondence: Meryl Theng <meryl.theng@unimelb.edu.au>

Table of contents

A ABC-SMC setup 2

A.1 Simulation process . . . 2

A.2 ABC-SMC algorithm . . . 3

A.3 Computational runtime and ABC-SMC convergence . . . 5

B Extended results 7

B.1 Forecast targets . . . 7

B.2 Additional runs . . . 7

B.2.1 Forecasts at earlier timepoint (2-weeks in) . . . 7

B.2.2 Forecasts for CCMN at later timepoints . . . 7

B.3 Spatial forecasts . . . 7

B.4 Posterior distributions . . . 14

B.4.1 Effect of premises density on local spread . . . 14

B.4.2 Shape of the spatial transmission kernel . . . 15

B.4.3 Background and baseline transmission rates . . . 15

B.4.4 Effect of vaccination . . . 15

B.4.5 Intra-premises transmission rate . . . 15

B.5 Forecast skill . . . 21

B.6 Vaccination . . . 25

References 30

A ABC-SMC setup

A.1 Simulation process

The general simulation process is as follows:

1. At the start of each simulated day of the epidemic, the states of all premises are updated to reflect the current knowledge of the system.
2. Values for unknown parameters are sampled from their respective priors.
3. The total amount of infection pressure in the system, τ_t, is calculated.

4. A random sample is drawn from a Poisson distribution with mean τ_t to calculate the number of new infections proposed to occur based on our expectation $E_t^* \sim \text{Pois}(\tau_t)$.
5. Proposed new infectees (E_t^*) are sampled from the set of those premises susceptible at that time, S_t, probabilistically weighted based on the infection pressure being exerted on them by all the infectious premises at that time, I_t.
6. The proposed infectors (I_t^*) of these new infectees are sampled from I_t probabilistically weighted based on the infection pressure that they are each exerting on each of the proposed infectees at time t.
7. The Poisson process is locally-thinned using a retrospective sampling technique that enables valid simulation of infection times across space and time (Karr, 1991). This involves only allowing each new infection to occur if the prevalence of infection on the proposed infector ($i \in I_t^*$) is greater than a random draw, u, from a uniform distribution $u \sim \mathcal{U}(0,1)$, i.e., if $h(t)_[i] > u$, then infection occurs at $j \in E_t^*$.
8. The states of all premises are updated, time is advanced a day ($t \to t+1$) and the process repeated from step 2.

A.2 ABC-SMC algorithm

Pseudo-code for our ABC-SMC algorithm are as follows (adapted from Minter and Retkute 2019):

1. Set the number of generations G, and the number of particles N_{px}.
2. Set the temporal and spatial tolerance values for each generation (temporal: $\epsilon_{\text{temp},1} > \epsilon_{\text{temp},2} > ..., > \epsilon_{\text{temp},G}$; spatial: $\epsilon_{\text{spat},1} < \epsilon_{\text{spat},2} < ..., < \epsilon_{\text{spat},G}$).
3. Set the generation indicator $g = 1$ and particle indicator $i = 1$.
4. If $g = 1$, sample θ^{**} from the prior distribution $P(\theta)$. If $g > 1$, sample θ^* from the previous generation θ_{g-1} with weights w_{g-1}, and perturb the particle to obtain $\theta^{**} \sim \mathcal{N}(\theta^*, \sqrt{2\tau_{g-1}^2})$.
5. Generate dataset D^{**} from the model using θ^{**} and calculate the temporal ($d_{\text{temp}}(D, D^{**})$) and spatial ($d_{\text{spat}}(D, D^{**})$) distances.
6. If $d_{\text{temp}}(D, D^{**}) \leq \epsilon_{\text{temp},g}$ and $d_{\text{spat}}(D, D^{**}) \geq \epsilon_{\text{spat},g}$, accept θ^{**}. Otherwise, reject and return to step 4.

7. Set $\theta_g^{(i)} = \theta^{**}$ and calculate the corresponding weight of the accepted particle i,

$$w_g^{(i)} = \begin{cases} \hat{P}(D|D^{**})P(\theta^{**}), & \text{if } g = 1. \\ \dfrac{\hat{P}(D|D^{**})P(\theta^{**})}{\sum_{i=1}^{N_{\text{px}}} w_{g-1} \cdot f_{\mathcal{N}}(\frac{\theta_g^{(i)} - \theta_{g-1}}{\sqrt{2\tau_{g-1}^2}} | \mu{=}0, \sigma{=}1)}, & \text{if } g > 1. \end{cases}$$

8. If $i < N_{\text{px}}$, make the increment $i = i + 1$ and go to step 4.
9. Normalise the weights so that $\sum_{k=1}^{N_{\text{px}}} w_g^{(i)} = 1$.
10. If $g < G$, set $g = g + 1$, go to step 3.

Note that the distance functions, sequence of tolerance values, number of generations, number of simulations for each parameter set, and perturbation kernel to use are user-defined choices that are context dependent (see Minter and Retkute 2019). These choices not only determine how efficiently the parameter space is explored, it can also affect the model's goodness-of-fit to the data.

Table A1: Parameter values for (i) the transmission model and (ii) the ABC-SMC.

	**Parameter(s)**	**Description**	**Value**
(i)	t_{detect}	Day of first detection/notification (indexed)	9, 10, 10, 12[a]
	t_{today}	Data date/time point	21, 35, 49 days after t_{detect}
	t_{lag}	Number of days before the current data date to fit model up to	3
	β_{intra}	Within-premises transmission rate	$\sim \mathcal{U}(1, 20)$
	σ_{intra}	Within-premises latency rate = 1/latent period	1/1
	γ_{intra}	Within-premises recovery rate = 1/infectious period	1/6
	α	Background transmission rate	$\sim \mathcal{U}(0, 0.02)$
	β_0	Baseline transmission rate (from S to E at premises-level)	$\sim \mathcal{U}(0, 0.02)$
	ψ	Kernel shape parameter (decay of # with distance between premises)	$\sim \mathcal{U}(0, 20)$
	ζ	Index of the count or density of animals on premises i at time t	$\sim \mathcal{U}(-2, 2)$
	ξ	Index of the count or density of animals on premises i at time t	$\sim \mathcal{U}(-2, 2)$
	θ	Effectiveness of vaccination	$\sim \mathcal{U}(0, 1)$
(ii)	N_{px}	Number of accepted particles	1,000

Parameter(s)	Description	Value
G	Maximum number of ABC-SMC generations/steps	8
$\epsilon_{\text{temp},1}, \epsilon_{\text{temp},2}, \ldots, \epsilon_{\text{temp},8}$	Temporal tolerance schedule (+/- daily case counts)	50, 40, 30, 20, 15, 10, 7.5, 6
$\epsilon_{\text{spat},1}, \epsilon_{\text{spat},2}, \ldots, \epsilon_{\text{spat},G}$	Spatial tolerance schedule (Pearson's correlation of grid cell case counts between observed and simulated data)	0.3, 0.4, 0.5, 0.6, 0.65, 0.675, 0.7, 0.725
$X \times X$	Number of grids (for grid cell case counts)	20×20

[a] Day 1 being the estimated date of first infection

A.3 Computational runtime and ABC-SMC convergence

Analyses were subject to a two-day runtime limit (including computing-cluster queue time) because, in some runs, later ABC–SMC generations stalled as tighter tolerances made new accepted particles increasingly difficult to obtain.

Observed runtimes varied with outbreak size, data volume, and parameter dimensionality. In the equine influenza case study, runtimes ranged from a few hours for the smallest cluster (Hunter Valley) to approximately 1–2 days for the largest clusters at later timepoints (Sydney Basin and CCMN), reflecting both model complexity and its spatially explicit formulation.

Across analyses, we observed diminishing returns beyond approximately generations 4–6, where runtime increased sharply with minimal gains in fit; in some cases, later generations yielded very few accepted particles within the time limit (e.g., Fig A1).

From an operational perspective, later generations are not required to generate useful forecasts. Once the fit stabilises, the most recent completed generation can be used to produce near-term projections, allowing results to be obtained within hours rather than days. Additional generations primarily tighten uncertainty around similar near-term inference rather than fundamentally altering forecast behaviour. This flexibility is consistent with real time decision support use, where timeliness may reasonably be prioritised over marginal gains in precision.

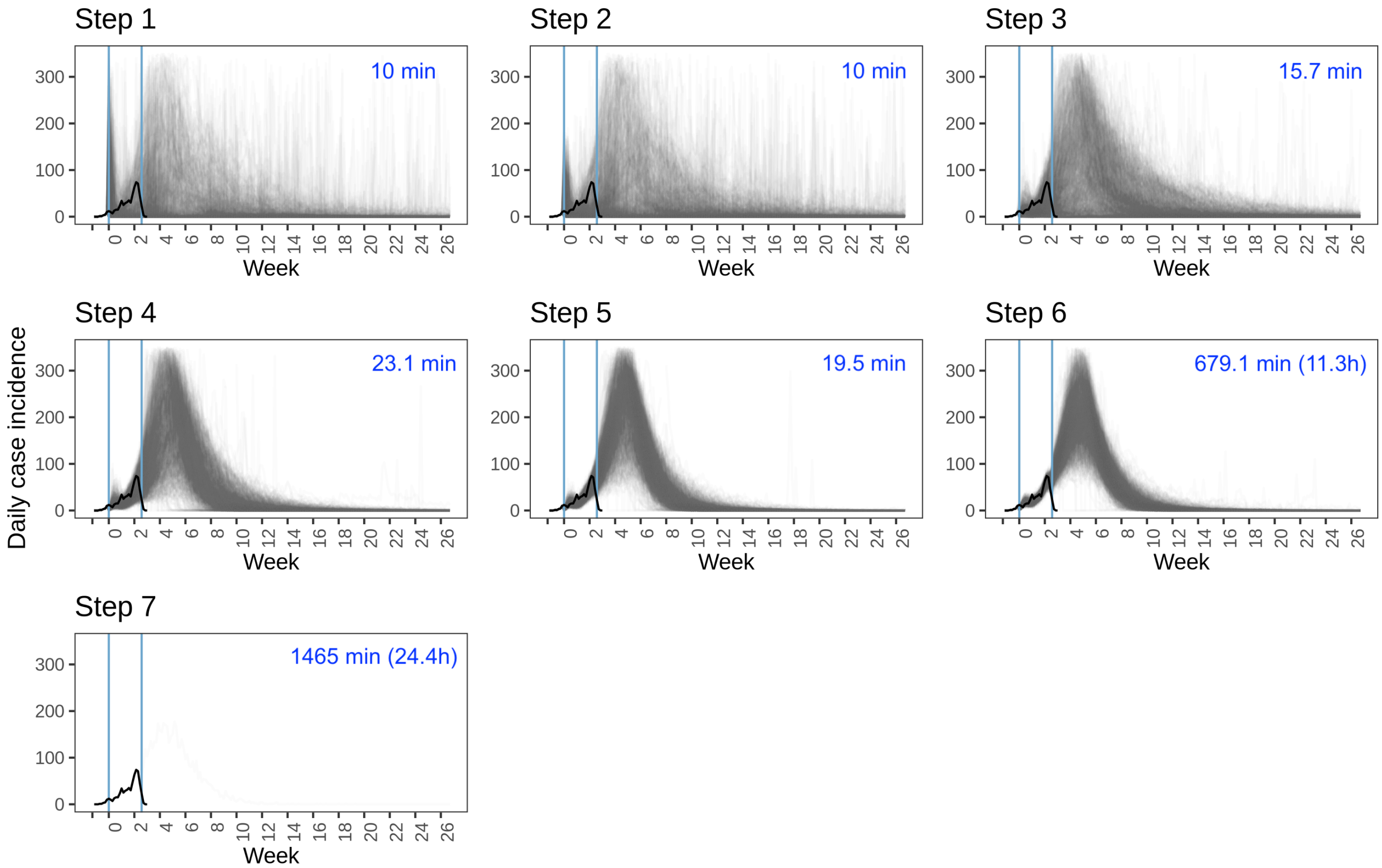

Fig A1: ABC-SMC step (generation) plots for the Sydney cluster at 3-weeks in. Panels show daily case-count trajectories (grey lines) simulated by accepted particles at each ABC-SMC generation, together with the time taken to complete each generation (including job-queue wait times). The black trend line shows the observed data available at 3-weeks, and light-blue vertical lines indicate the fitting window. Note that at generation 7 only a single particle was accepted within the 24-hour runtime limit.

B Extended results

B.1 Forecast targets

Forecasts of summary statistics by cluster and time point are given in Table B1.

B.2 Additional runs

B.2.1 Forecasts at earlier timepoint (2-weeks in)

In our post-hoc analysis, we produced forecasts for an additional, earlier timepoint in the ascending phase of the outbreak (2-weeks in; Fig B1). At this early stage, the lag in reporting was substantial, and fitting to this incomplete data led to (i) forecasts with even wider uncertainty intervals (compared to forecasts at 3-weeks in), and (ii) an underestimation of outbreak size and severity in clusters where daily caseloads were low (CCMN, Tamworth, Hunter), or a much delayed outbreak peak (Sydney).

B.2.2 Forecasts for CCMN at later timepoints

We produced forecasts for the Central Coast-Maitland-Newcastle (CCMN) cluster at two additional timepoints beyond the observed peak (9- and 11-weeks in). Fig B2 shows that though these forecasts no longer predicted a much larger outbreak, the model struggled to fit the observed outbreak and predicted a relatively smaller but protracted outbreak. Our model could not find simulated outbreaks that closely matched the observations, likely due to the multiple changes to the system (control policies, etc.) that were not modelled. In such cases, we recommend starting the model fit from a later date (i.e., aligned with the most recent policy change) and initialising simulations using data observed prior to that date. In the future, we also aim to explore how to incorporate time-varying parameters to better capture such changes in the system.

B.3 Spatial forecasts

Spatial forecasts for the CCMN, Tamworth and Hunter Valley regions can be found in following Figs B3–B5.

Table B1: Predictions of summary statistics by cluster. Median estimates accompanied by their 95% credible intervals (in brackets) are provided. Summary statistics predicted were: final outbreak size ('final_size'), timing of peak ('peak_t'), case count at peak ('peak_n'), and outbreak duration ('duration').

(a) Sydney Basin

	Predicted (3 weeks)	Predicted (5 weeks)	Predicted (7 weeks)	Observed
final_size	6702 (4162,7350)	3568 (2795,6243)	3054 (2649,3467)	2875
peak_t	42 (36,49)	37 (24,57)	31 (24,39)	26
peak_n	230 (121,382)	98 (71,132)	96 (72,122)	98
duration	125 (83,188)	154 (117,196)	135 (103,185)	126

(b) Central Coast-Maitland-Newcastle

	Predicted (3 weeks)	Predicted (5 weeks)	Predicted (7 weeks)	Observed
final_size	948 (9,6548)	5268 (3929,6157)	5160 (3628,6052)	1292
peak_t	40 (7,166)	71 (59,92)	78 (63,97)	53
peak_n	57 (3,6471)	132 (75,219)	100 (55,160)	48
duration	77 (12,185)	176 (134,185)	184 (165,185)	128

(c) Tamworth

	Predicted (3 weeks)	Predicted (5 weeks)	Predicted (7 weeks)	Observed
final_size	1366 (61,2317)	1202 (652,2013)	912 (524,1598)	582
peak_t	58 (12,130)	52 (30,76)	41 (24,94)	31
peak_n	34 (5,768)	30 (21,57)	22 (16,34)	29
duration	153 (45,202)	160 (106,202)	178 (101,202)	106

(d) Hunter Valley

	Predicted (3 weeks)	Predicted (5 weeks)	Predicted (7 weeks)	Observed
final_size	243 (100,1061)	276 (212,365)	306 (230,794)	404
peak_t	35 (11,76)	31 (24,45)	36 (23,63)	31
peak_n	11 (5,70)	14 (10,19)	15 (9,23)	21
duration	99 (66,189)	91 (69,138)	98 (73,188)	105

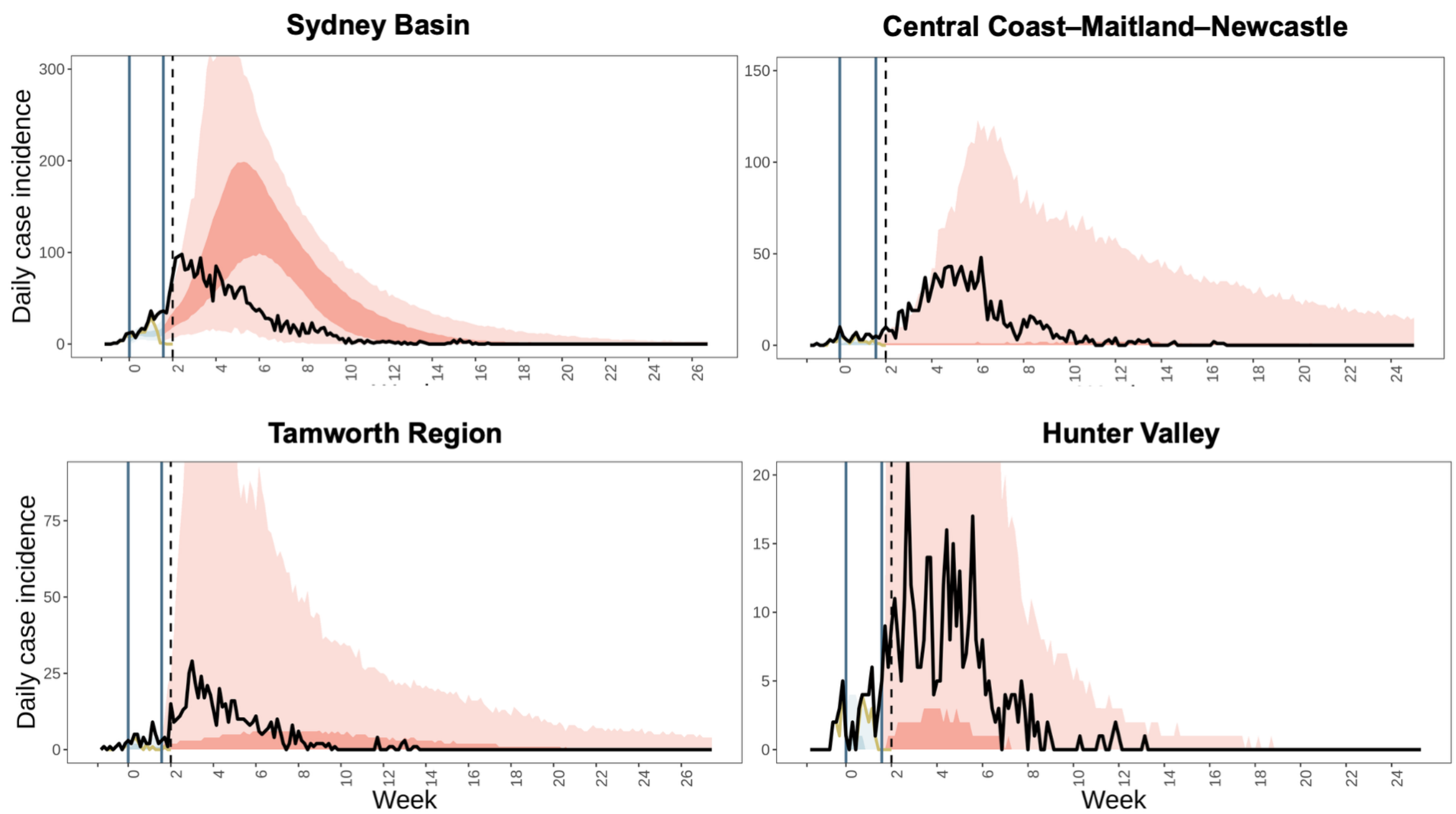

Fig B1: Forecast of daily EI case incidence for each cluster at 2-weeks after the first case detection. The blue shaded regions represent model fit to the data available at the time of forecasting (dark yellow lines). The red shaded regions represent the forecast. Vertical black dashed lines indicate the time point. Vertical blue lines indicate the time window of data the model was fit to (accounting for delay with a 3-day lag). Black lines represent all reported daily case counts (i.e., all observations by end of outbreak). Shaded areas indicate the 50% and 95% percentile of 1000 simulated trajectories (of accepted particles).

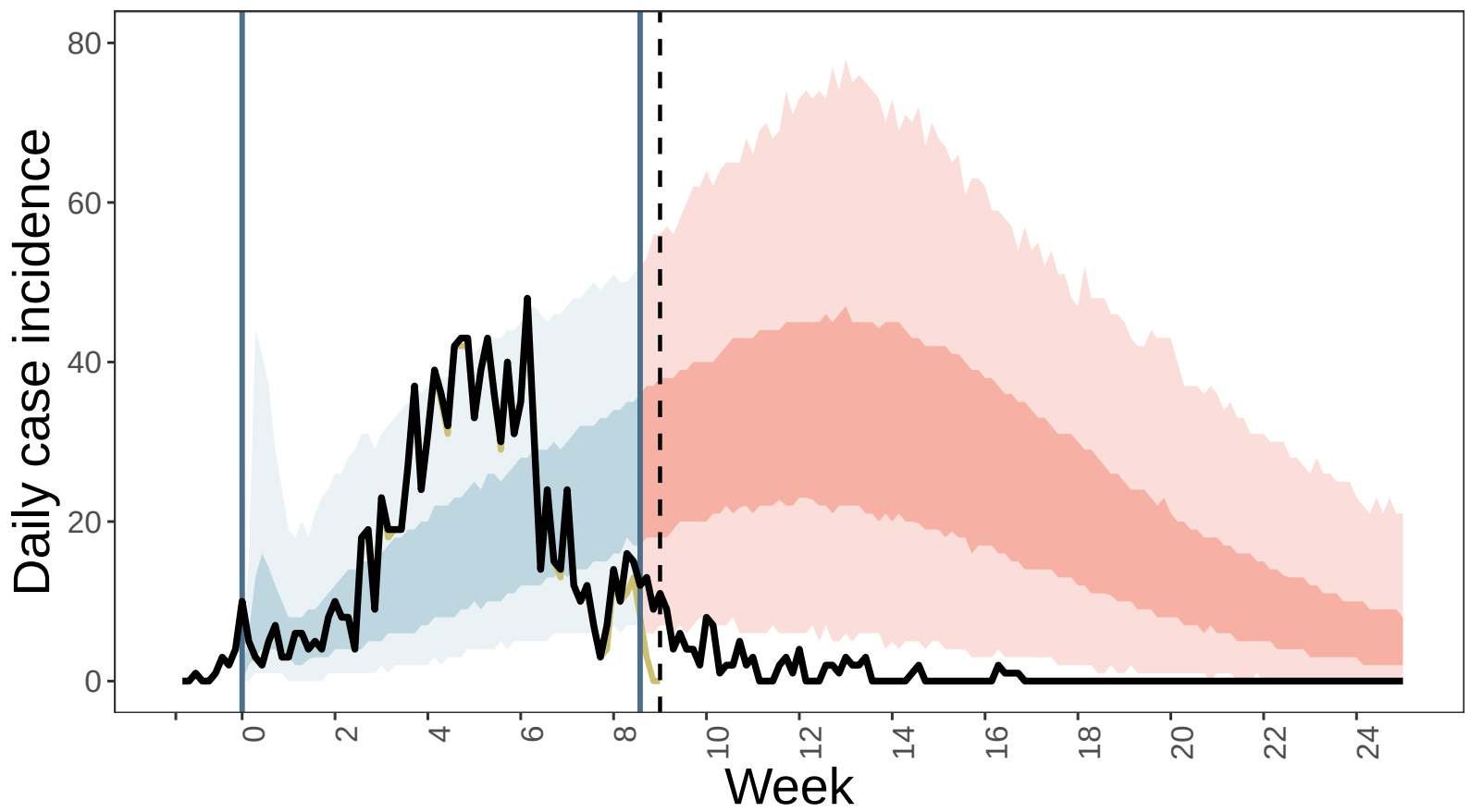

(a)

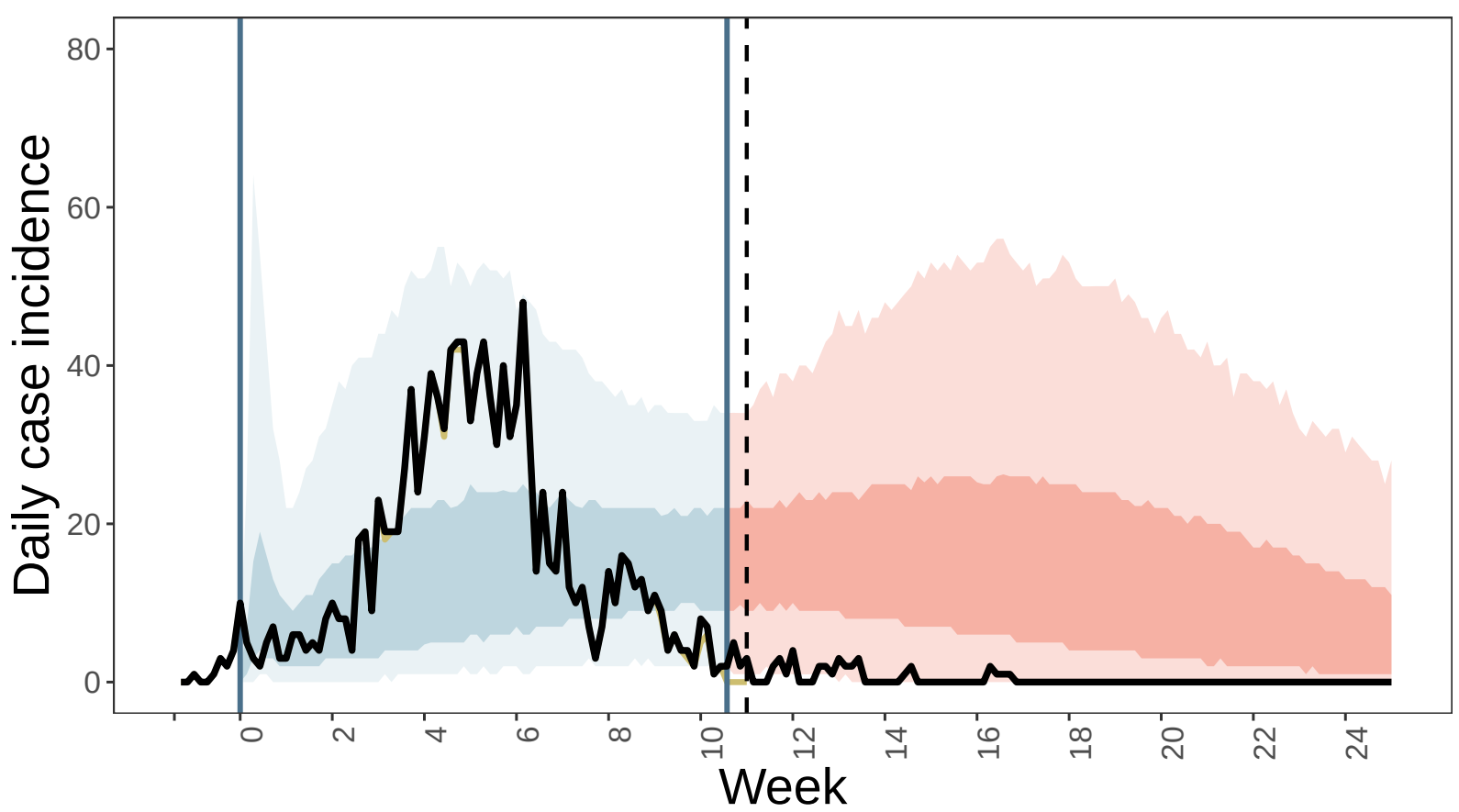

(b)

Fig B2: Forecast of daily EI case incidence for the Central Coast-Maitland-Newcastle (CCMN) cluster at two additional timepoints beyond the observed peak: (a) 9-weeks in, and (b) 11-weeks in. The blue shaded regions represent model fit to the data available at the time of forecasting (dark yellow lines). The red shaded regions represent the forecast. Vertical black dashed lines indicate the time point. Vertical blue lines indicate the time window of data the model was fit to (accounting for delay with a 3-day lag). Black lines represent all reported daily case counts (i.e., all observations by end of outbreak). Shaded areas indicate the 50% and 95% percentile of 1000 simulated trajectories (of accepted particles).

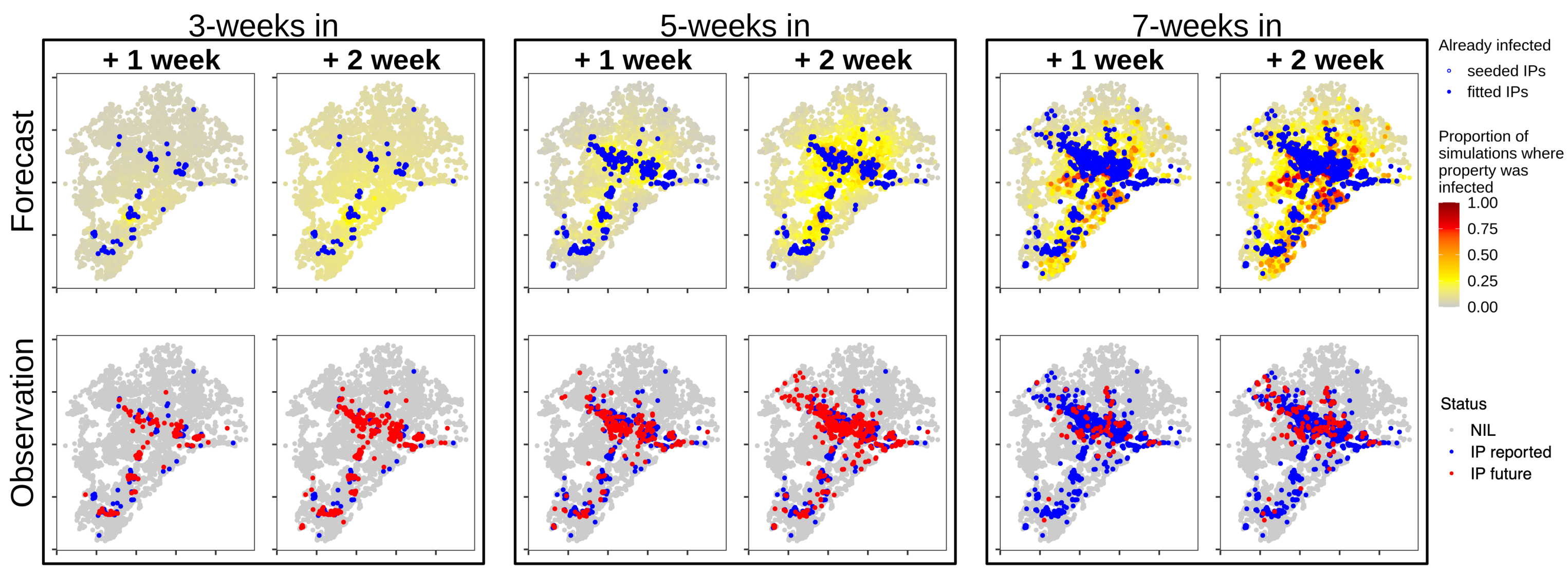

Fig B3: Comparison of simulated and observed outbreaks for the **Central Coast-Maitland-Newcastle** region for each timepoint. Spatial maps showing proportion of runs (out of 1000 simulation runs) in which a premises was infected between the date of first detection and 1-week and 2-weeks ahead from the timepoint (top panel); compared to the single observed realisation of the outbreak at the same respective dates (bottom panel; "NIL": non-infectious premises; "IP reported": infectious premises reported before the timepoint; "IP future": infectious premises reported after the timepoint).

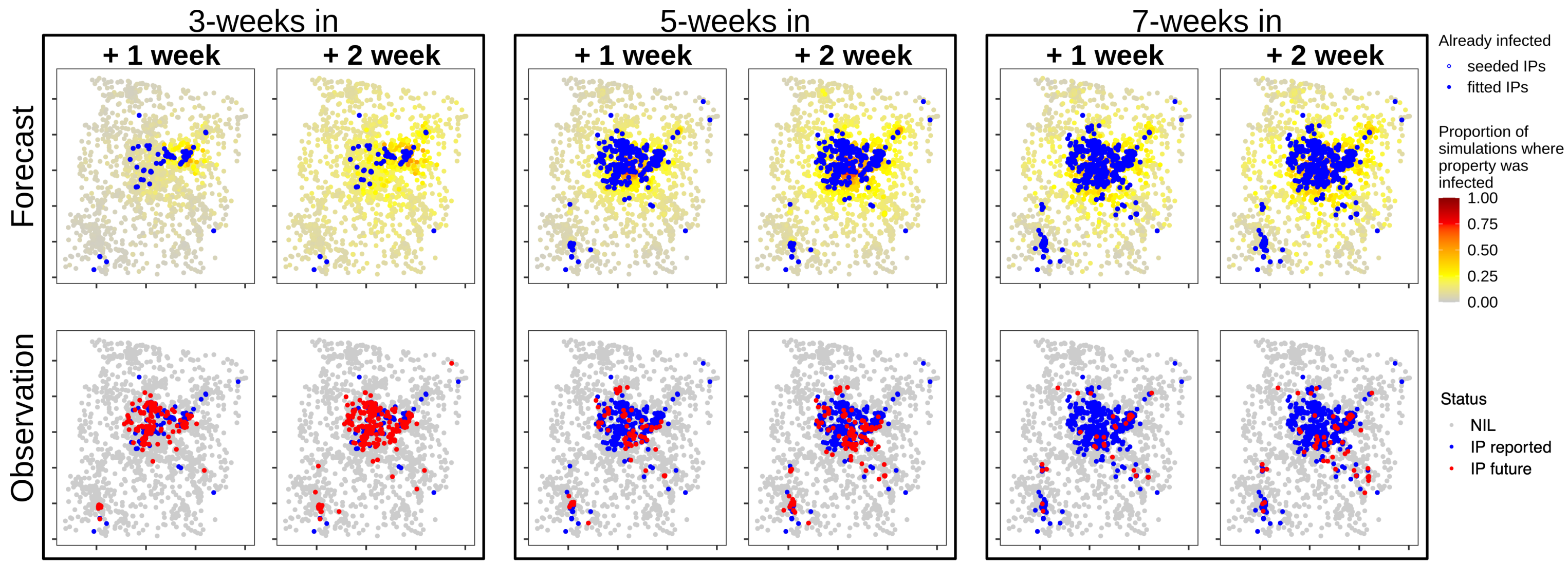

Fig B4: Comparison of simulated and observed outbreaks for the **Tamworth** region for each timepoint. Spatial maps showing proportion of runs (out of 1000 simulation runs) in which a premises was infected between the date of first detection and 1-week and 2-weeks ahead from the timepoint (top panel); compared to the single observed realisation of the outbreak at the same respective dates (bottom panel; "NIL": non-infectious premises; "IP reported": infectious premises reported before the timepoint; "IP future": infectious premises reported after the timepoint).

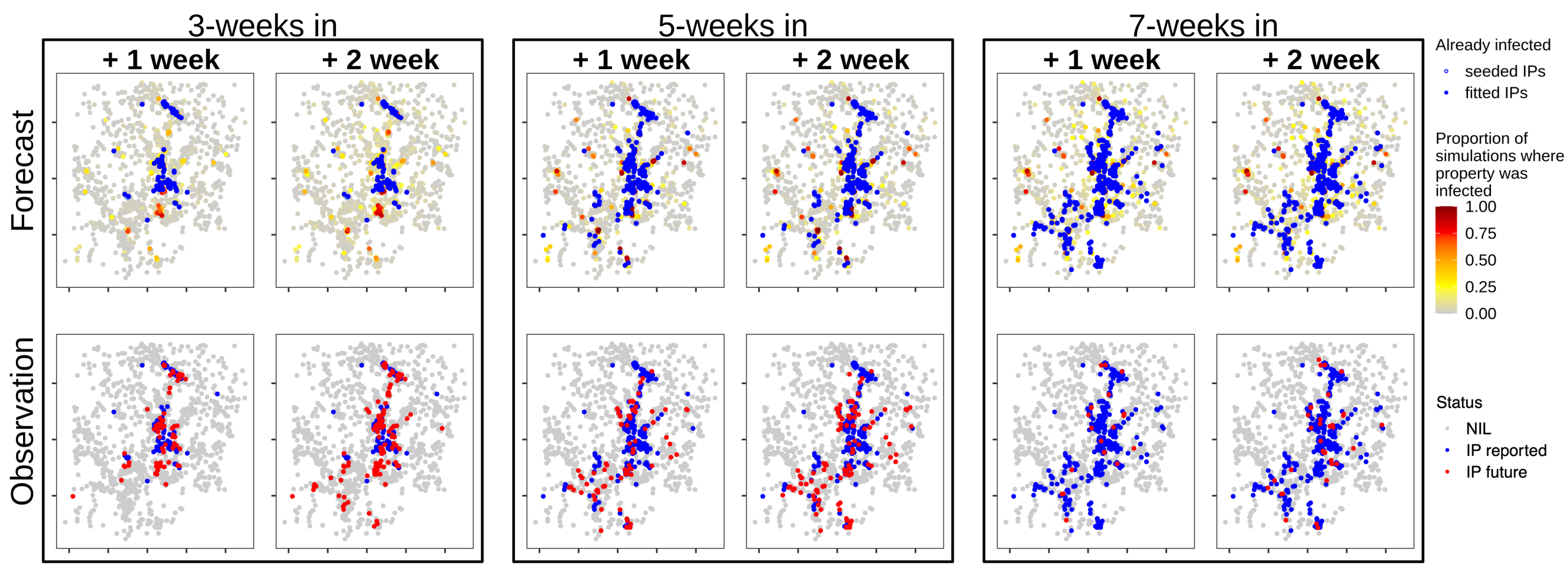

Fig B5: Comparison of simulated and observed outbreaks for the **Hunter Valley** region for each timepoint. Spatial maps showing proportion of runs (out of 1000 simulation runs) in which a premises was infected between the date of first detection and 1-week and 2-weeks ahead from the timepoint (top panel); compared to the single observed realisation of the outbreak at the same respective dates (bottom panel; "NIL": non-infectious premises; "IP reported": infectious premises reported before the timepoint; "IP future": infectious premises reported after the timepoint).

B.4 Posterior distributions

Posterior distributions of each inferred model parameter at each timepoint (3-, 5- and 7-weeks in) are presented by cluster: Sydney (Fig B6), CCMN (Fig B7), Tamworth (Fig B8), and Hunter Valley (Fig B9). Note that we have also included posterior distributions recovered from the full dataset (i.e., $t_{\text{today}} = t_{\max} - 1$) for each cluster.

B.4.1 Effect of premises density on local spread

Parameters on the non-linear effect of premises density on local spread (ξ, ζ) appeared to be the most sensitive, with posterior estimates diverging the most from their uniform priors and increased in precision through time across all clusters. Moreover, these estimates varied in both direction and strength between clusters.

Posterior estimates for ξ at timepoint 3 indicated that higher density premises were much less susceptible compared to lower density premises in the Sydney cluster ($\xi = -1.46[-1.71, -1.17]$; e.g., a premises of 10 horses/ha was 29 times *less* likely to be infected than a premises with 1 horse/ha), but were more susceptible than lower density premises in the CCMN ($\xi = 0.35[-0.45, 0.68]$; e.g., a premises of 10 horses/ha was 2.2 times *more* likely to be infected than a premises with 1 horses/ha) and Hunter Valley ($\xi = 0.8[0.1, 1.1]$; e.g., a premises of 10 horses/ha was 6.3 times *more* likely to be infected than a premises with 1 horse/ha) cluster. Density had a negligible effect on premises susceptibility in the Tamworth cluster ($\xi = -0.09[-0.85, 0.58]$).

Posterior estimates for ζ at timepoint 3 indicated that higher density premises were more infectious compared to lower density premises in the Tamworth ($\zeta = 0.6[-0.82, 1.15]$) and Hunter valley ($\zeta = 0.32[-0.52, 0.65]$) cluster, where a premises of 10 times the density would be 4 and 2.1 times *more* likely to infect other premises respectively. Conversely, higher density premises were less infectious compared to lower density premises in the CCMN cluster ($\zeta = -0.58[-1.16, 0.61]$), where a premises of 10 times the density would be 3.8 times *less* likely to infect other premises. Density had a negligible effect on premises infectivity in the Sydney cluster ($\xi = -0.06[-0.44, 0.37]$).

B.4.2 Shape of the spatial transmission kernel

Posterior distributions of spatial kernel shape (ψ) diverged from their flat prior distributions in the CCMN and Tamworth clusters, but estimates were generally imprecise and did not improve through time (4.36[0.90,17.68] and 4.07[0.32,17.72] at timepoint 3 respectively). No divergence from the prior was observed in the Sydney and Hunter Valley cluster.

B.4.3 Background and baseline transmission rates

There was no learning (i.e., posterior distribution diverging from the prior)on background spread rate (α) in all clusters and timepoints.

The amount of learning observed in the baseline transmission rate (β_0) appeared to positively correlate with the size of the EI cluster. Posterior estimates in the Sydney cluster were the most precise, followed by the CCMN, and in the smallest clusters (Tamworth and Hunter Valley), were very imprecise. Despite these regional differences, a trend of increasing precision in estimates and decreasing median estimates was observed across all clusters,

B.4.4 Effect of vaccination

Posterior distributions of the vaccine effectiveness parameter (θ) did not diverge from its flat prior across all clusters and timepoints. This was also observed in subsequent model fits to the full dataset (i.e., $t_{\text{today}} = t_{\max} - 1$), though some information gain (relative to the prior) was observed only in the heavily vaccinated CCMN cluster ($\theta = 0.69 = [0.09, 0.99]$). See Section B.6 for further details.

B.4.5 Intra-premises transmission rate

Posterior estimates on intra-premises transmission rate (β_{intra}) diverged from its wide uniform prior ($\mathcal{U}(1, 20)$) but were generally similar across timepoints and clusters (95% CrI $\approxeq [1, 5]$). Corresponding estimates of $R0_{\text{intra}}$ are provided in Table B2, calculated from posterior estimates of β_{intra} (i.e., $R0_{\text{intra}} = \beta_{\text{intra}} \cdot 1/\gamma_{\text{intra}}$, where $\gamma_{\text{intra}} = 1/6$).

Table B2: Estimates of $R0_{\text{intra}}$ by cluster and timepoint, calculated from posterior estimates of β_{intra} (see text for details). Median estimates accompanied by their 95% credible intervals (in brackets) are provided.

	3-weeks in	5-weeks in	7-weeks in
Sydney	15.98 [6.59,28.76]	16.79 [6.54,28.74]	15.84 [6.51,28.79]
CCMN	22.78 [7.07,71.2]	19.04 [7.24,29.3]	16.87 [6.74,29.1]
Tamworth	18.33 [6.98,29.71]	16.53 [6.56,28.59]	16.43 [6.56,28.77]
Hunter	17.55 [6.51,29.08]	16.51 [6.8,28.9]	16.76 [6.63,28.87]

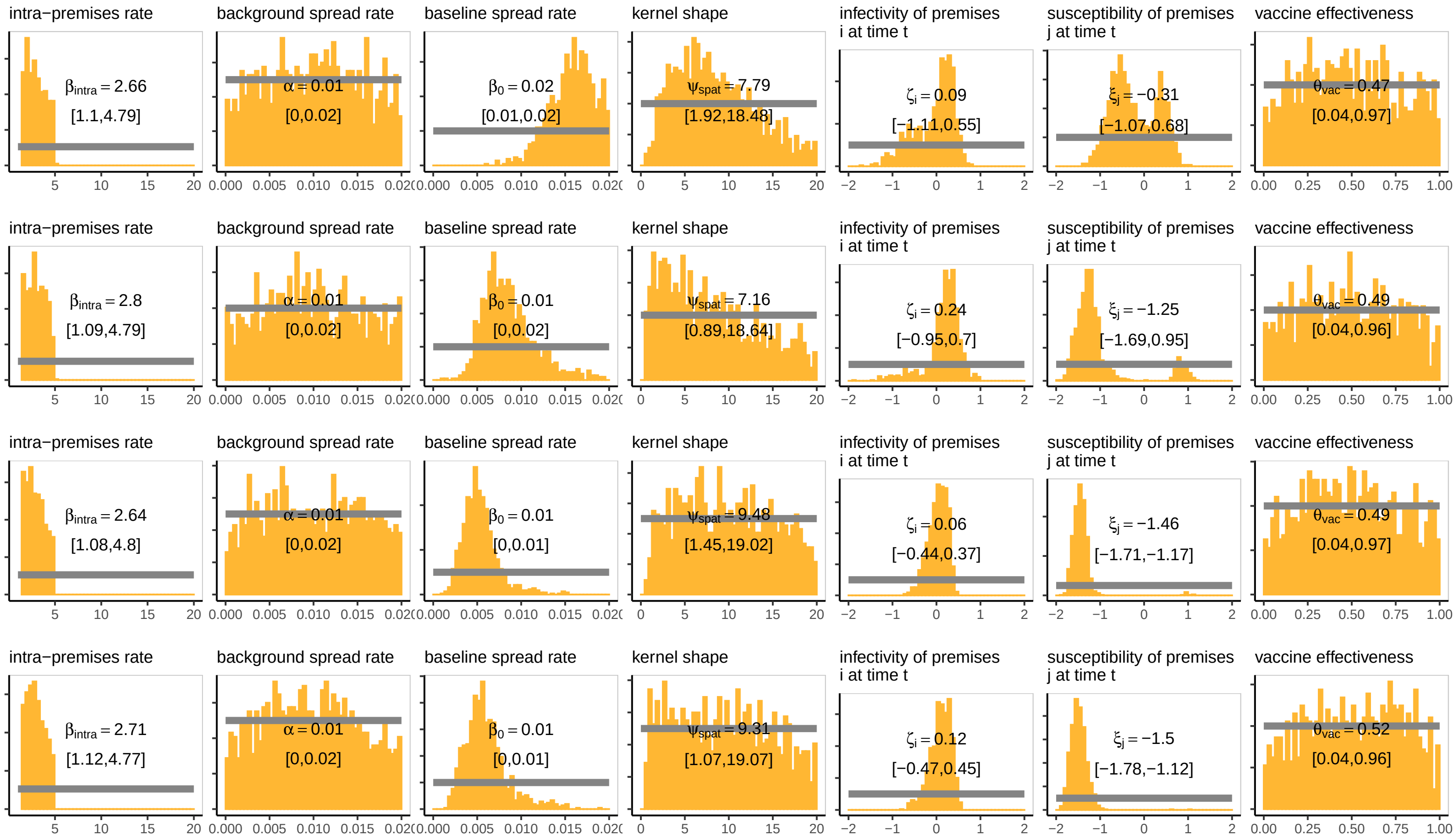

Fig B6: Prior (grey) and posterior (orange) distributions for all unknown model parameters based on data available at all three timepoints (3-, 5-, 7-weeks in) and at the end of the outbreak (bottom panel) for the *Sydney Basin region*.

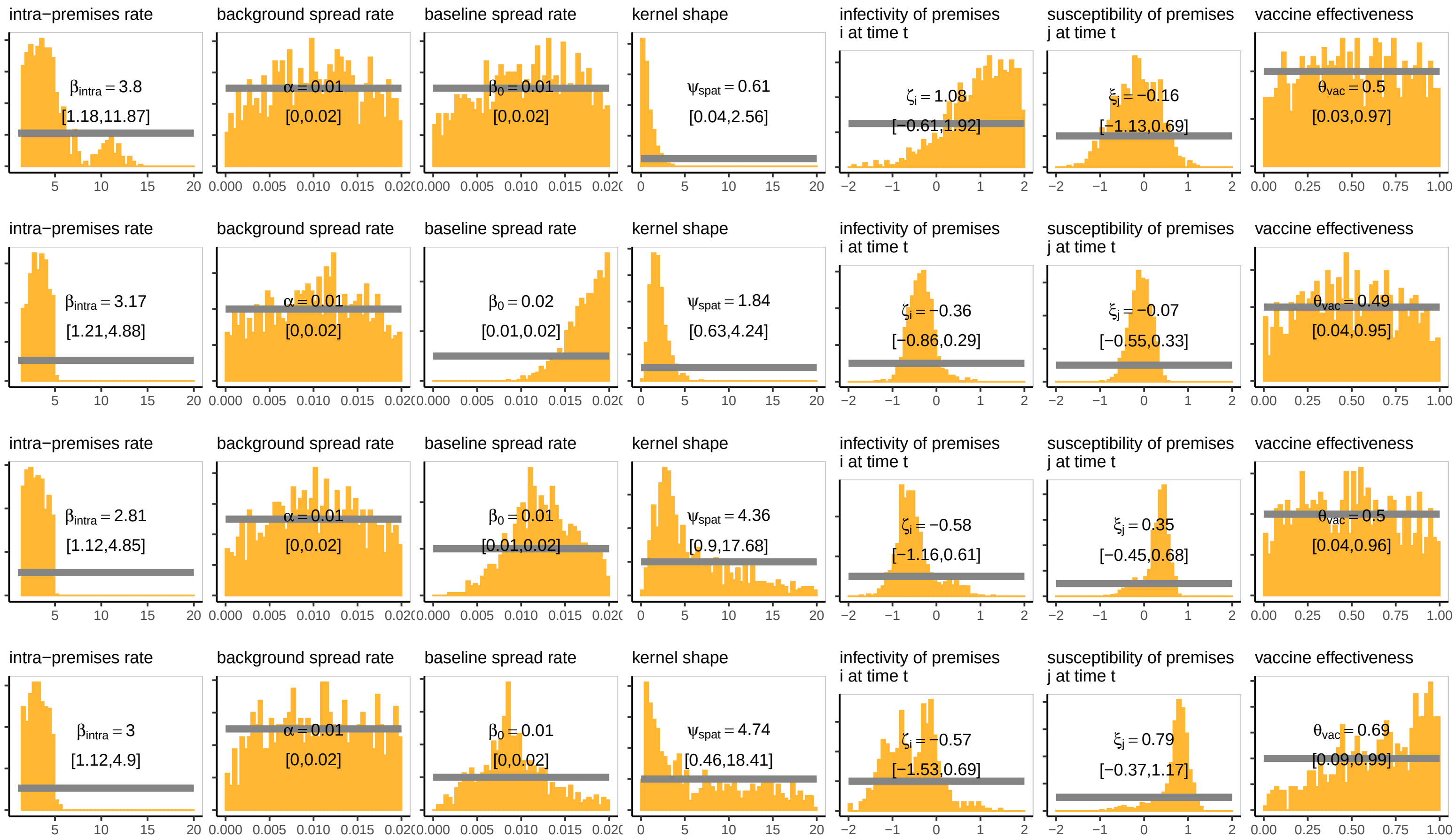

Fig B7: Prior (grey) and posterior (orange) distributions for all unknown model parameters based on data available at all three timepoints (3-, 5-, 7-weeks in) and at the end of the outbreak (bottom panel) for the *Central Coast-Maitland-Newcastle region.*

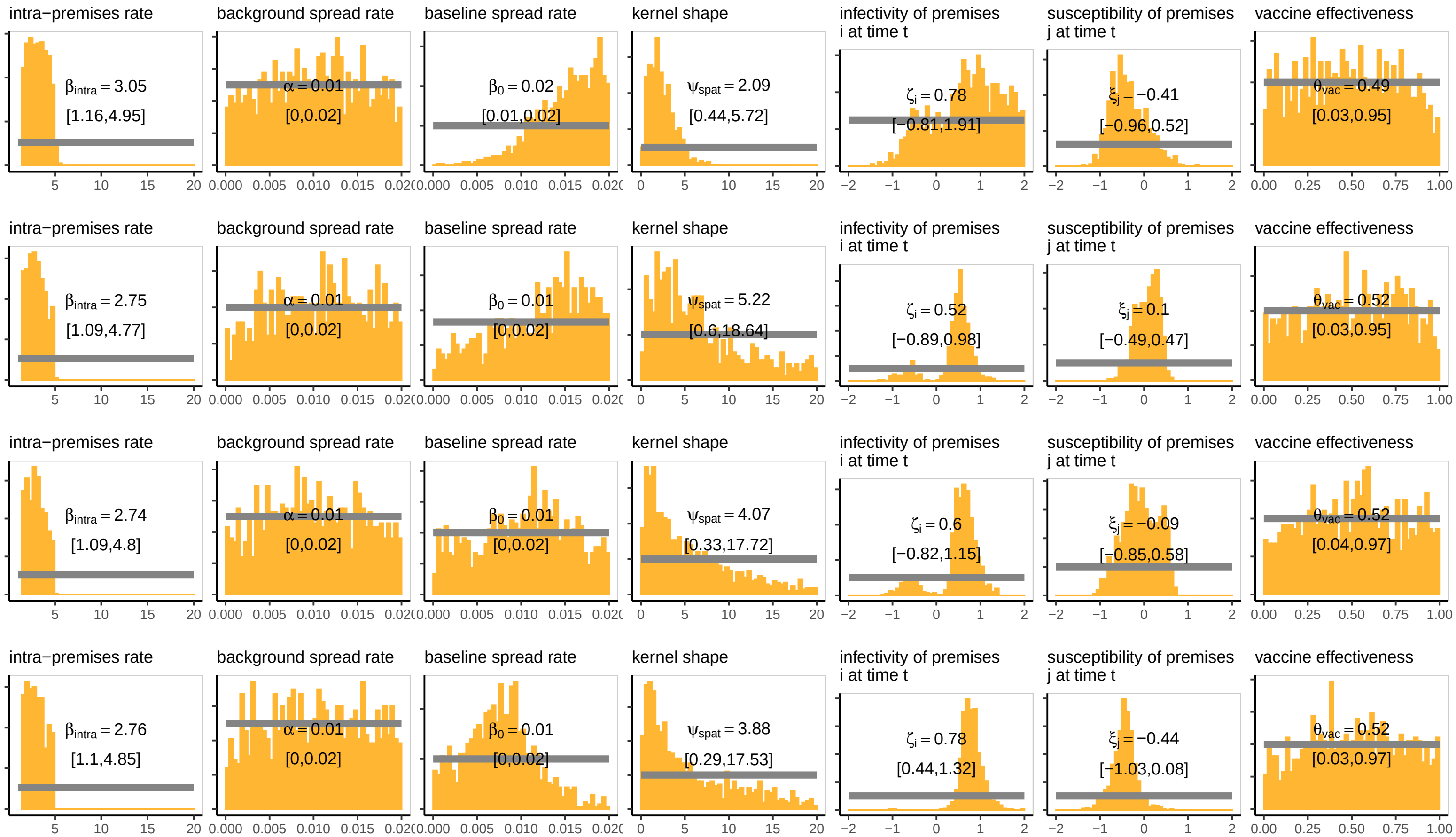

Fig B8: Prior (grey) and posterior (orange) distributions for all unknown model parameters based on data available at all three timepoints (3-, 5-, 7-weeks in) and at the end of the outbreak (bottom panel) for the *Tamworth region*.

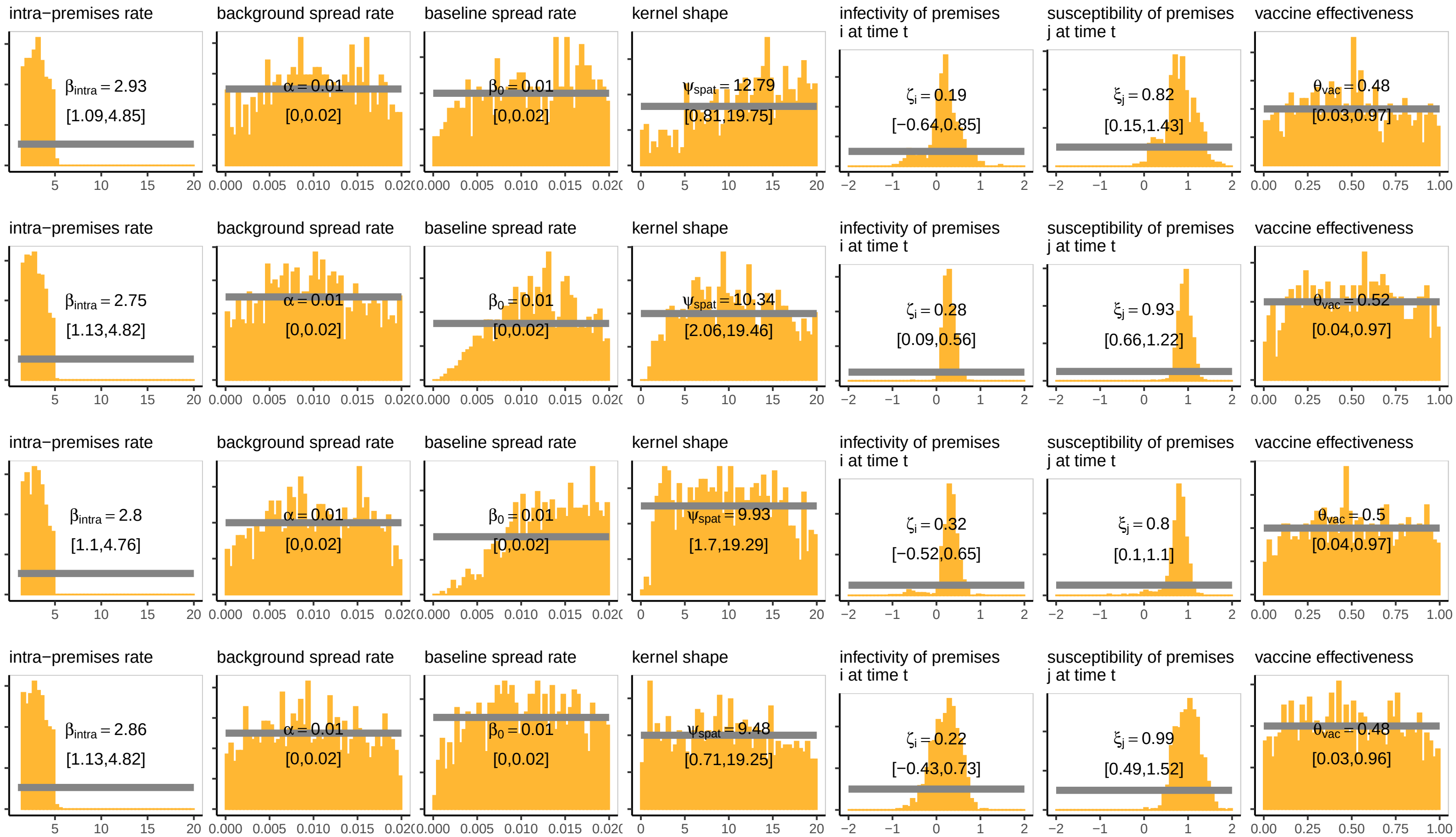

Fig B9: Prior (grey) and posterior (orange) distributions for all unknown model parameters based on data available at all three timepoints (3-, 5-, 7-weeks in) and at the end of the outbreak (bottom panel) for the *Hunter Valley region*.

B.5 Forecast skill

Fig B10 shows forecast skill by lead time for each of the four EI outbreak clusters.

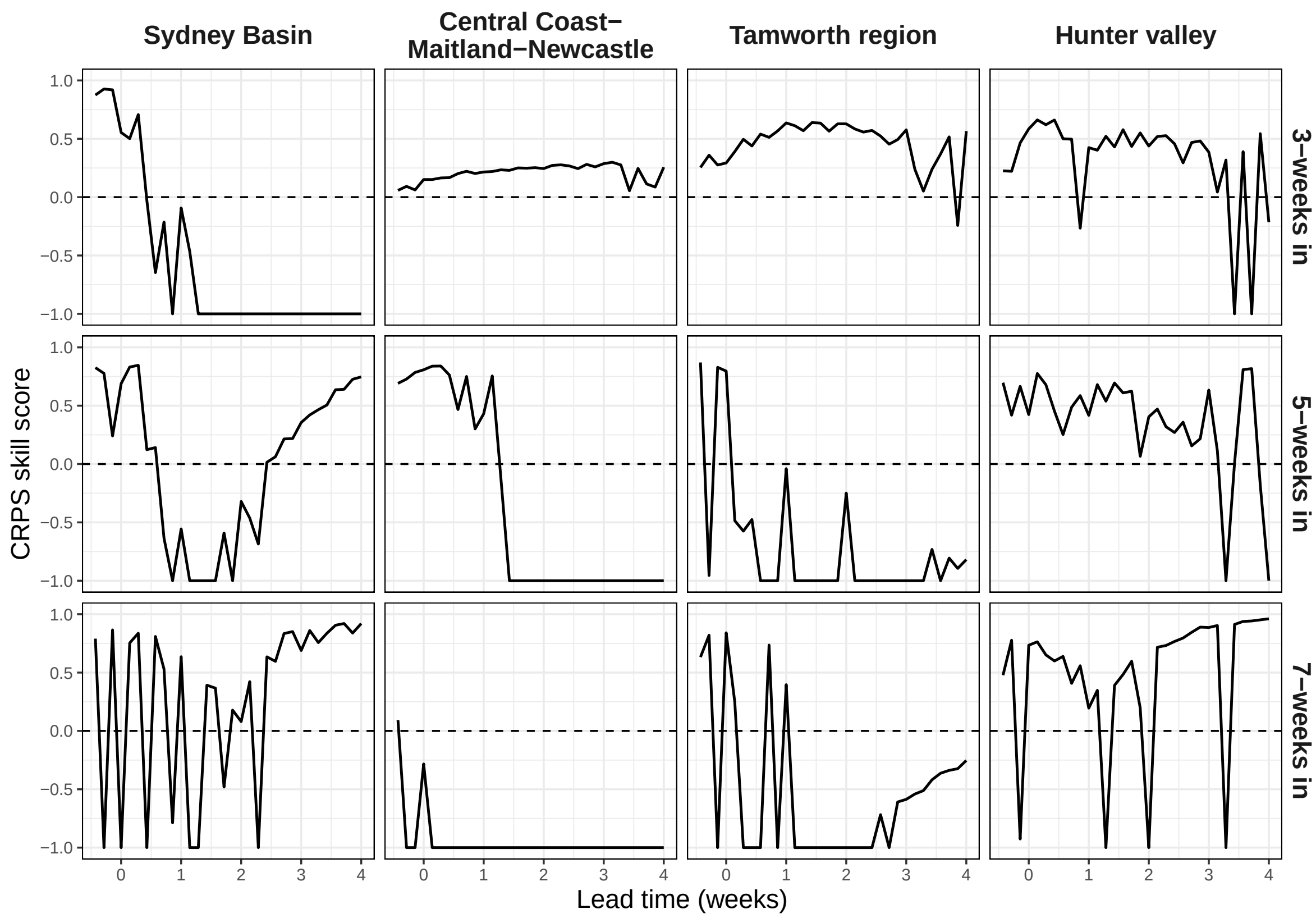

Fig B10: Forecast skill of EI daily case counts by lead time for four outbreak clusters in Australia, shown separately for each time point (right-hand axis labels). Positive skill scores indicate that the forecasts outperformed the naive benchmark, with a maximum possible score of one (i.e., perfect accuracy and no uncertainty). Negative skill scores indicate underperformance relative to the naive benchmark, with a mininum score of $-\infty$. Skill scores below -1 were truncated.

We also considered forecast skill as a function of the number of cases ultimately recorded on each day in each cluster (comprising 384 observations). We divided the daily case counts into four bins: 0 cases (n = 36, 9.4%); 1–4 cases (n = 78, 20.3%); 5–9 cases (n = 69, 18.0%); 10–29 cases (n = 129, 33.6%); and $\geq$ 30 cases (n = 72, 18.8%). There appeared to be no consistent trend between forecast skill and daily case numbers across the four clusters (Fig B11).

Spatial forecast skill generally improved after the first timepoint (3-weeks in), and tended to

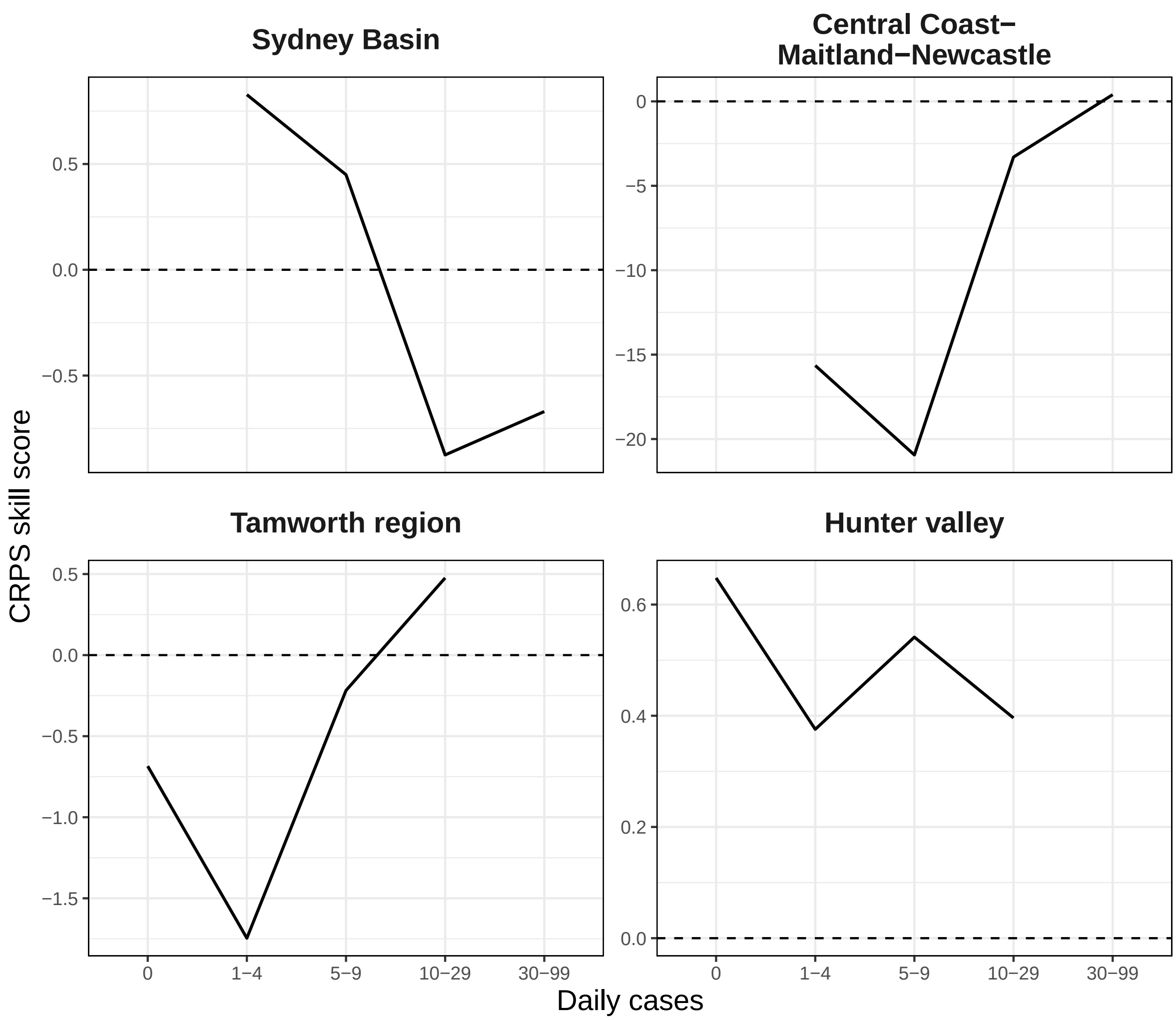

Fig B11: CRPS skill scores binned by the number of cases ultimately recorded for the day of prediction shown separately for each outbreak cluster.

be higher for 2-weeks ahead projections (Fig B12).

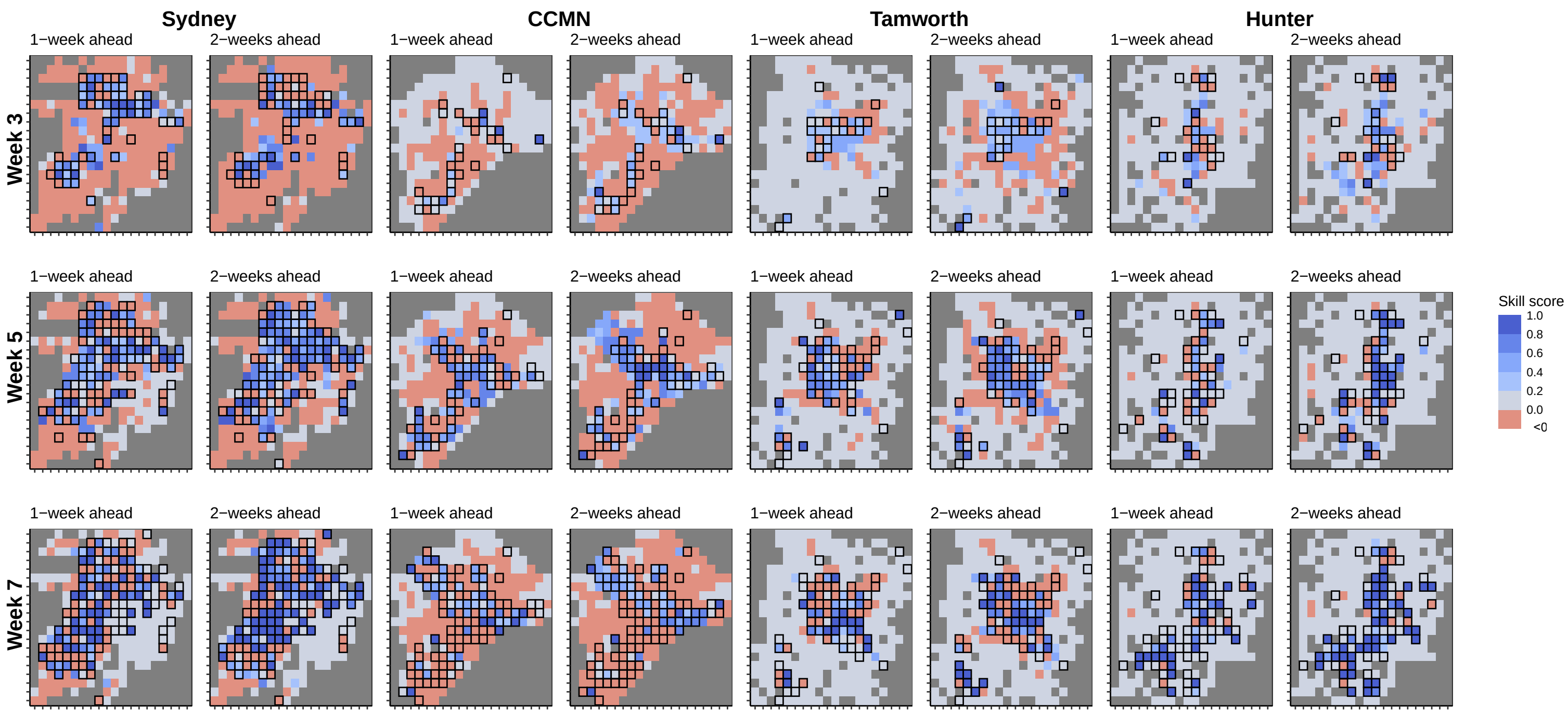

Fig B12: Spatial forecast skill for 1- and 2-week ahead case counts for each cluster and timepoint. Black outlines denote grids within the active outbreak zone (i.e., observed case counts >0).

B.6 Vaccination

We provided an initial demonstration through our 2007 EI outbreak case study where we evaluated the impact of the implemented vaccination policy through our vaccine effectiveness parameter θ (Section B.4.4).

To have a noticeable effect at the population-level, the vaccination rate would have to outpace the infection rate (Probert 2018); though in local clusters within disease-free premises, they can have a protective effect. The lack of information gained relative to the prior for θ suggests that the implemented vaccination policy did little to contain outbreak, corroborating the findings of other analyses of the 2007 EI outbreak (Garner et al. 2011; Firestone 2012). It did not matter that substantial vaccinations were occurring because they occurred in the peripheral regions of each cluster and when the outbreak was already dying out (Fig B13, Fig B14, Fig B15, Fig B16). Furthermore, peak immunity took around 14 days to reach (Edlund Toulemonde et al. 2005). Though, the model fit to the full dataset provided some indication that vaccinations had some protective effect only in the CCMN cluster and could have prevented a second wave from occuring, possibly due to a large proportion of the cluster being vaccinated. These findings are preliminary and should be investigated further in future work.

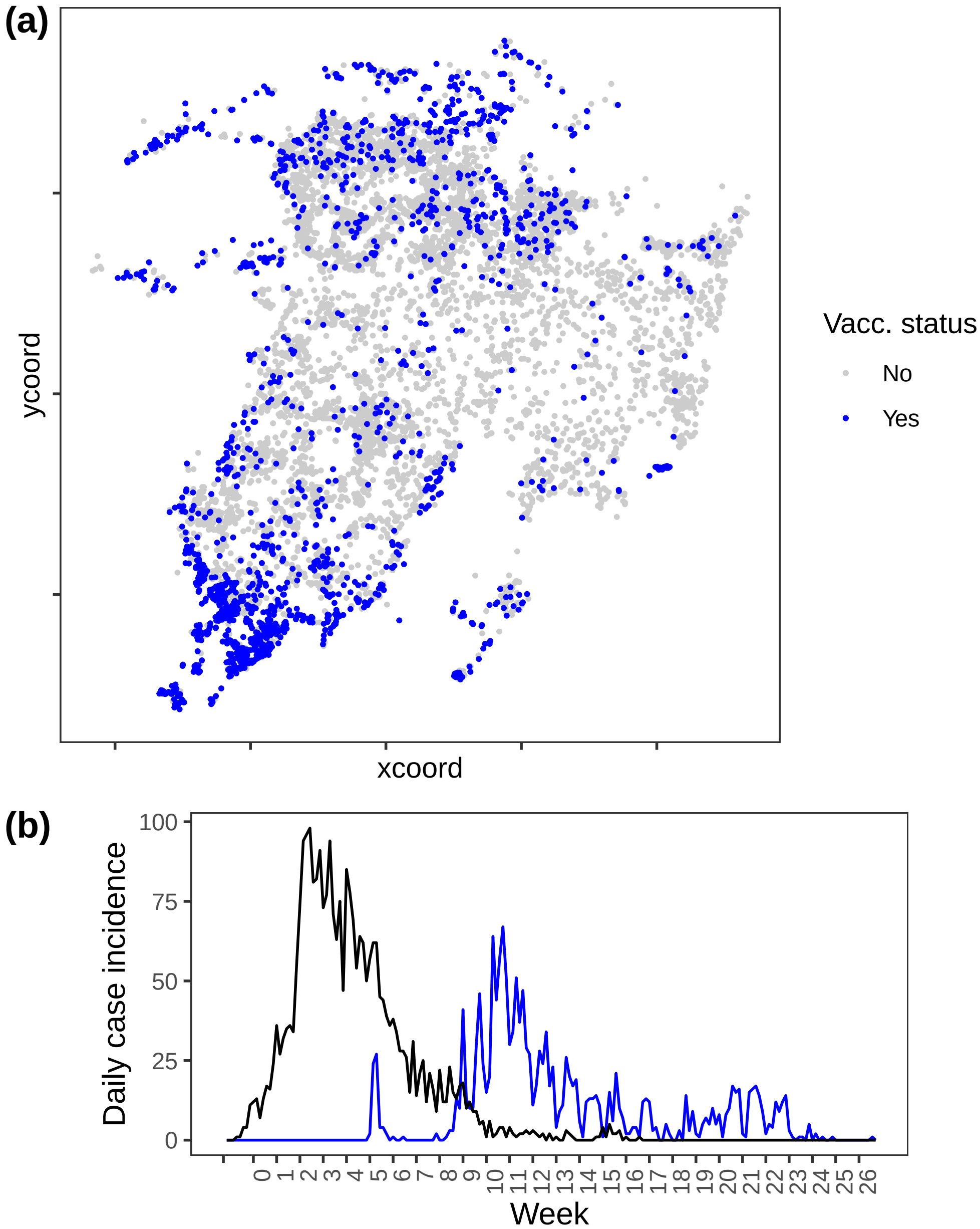

Fig B13: Vaccinations in the *Sydney region*.

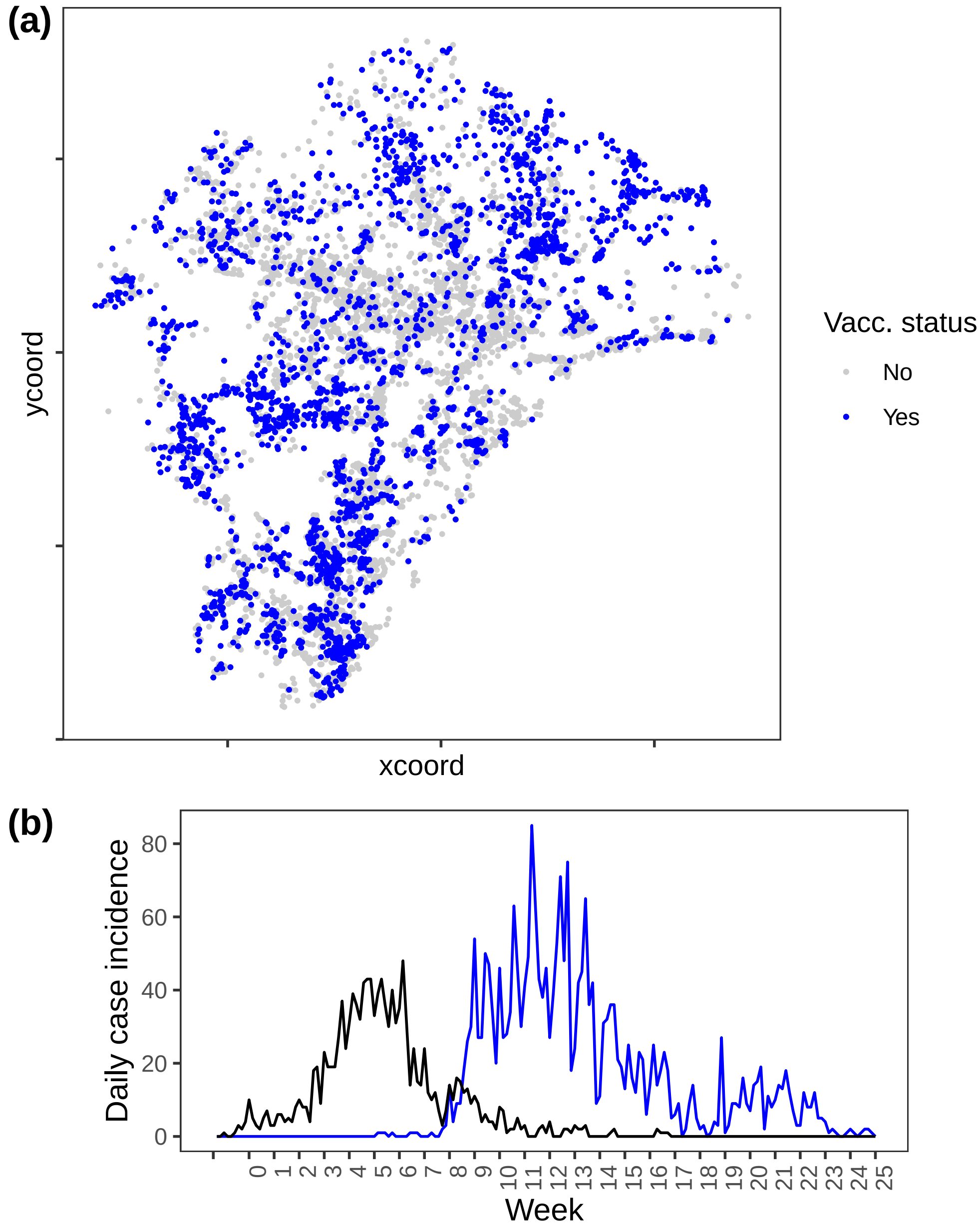

Fig B14: Vaccinations in the *Central Coast-Maitland-Newcastle region*.

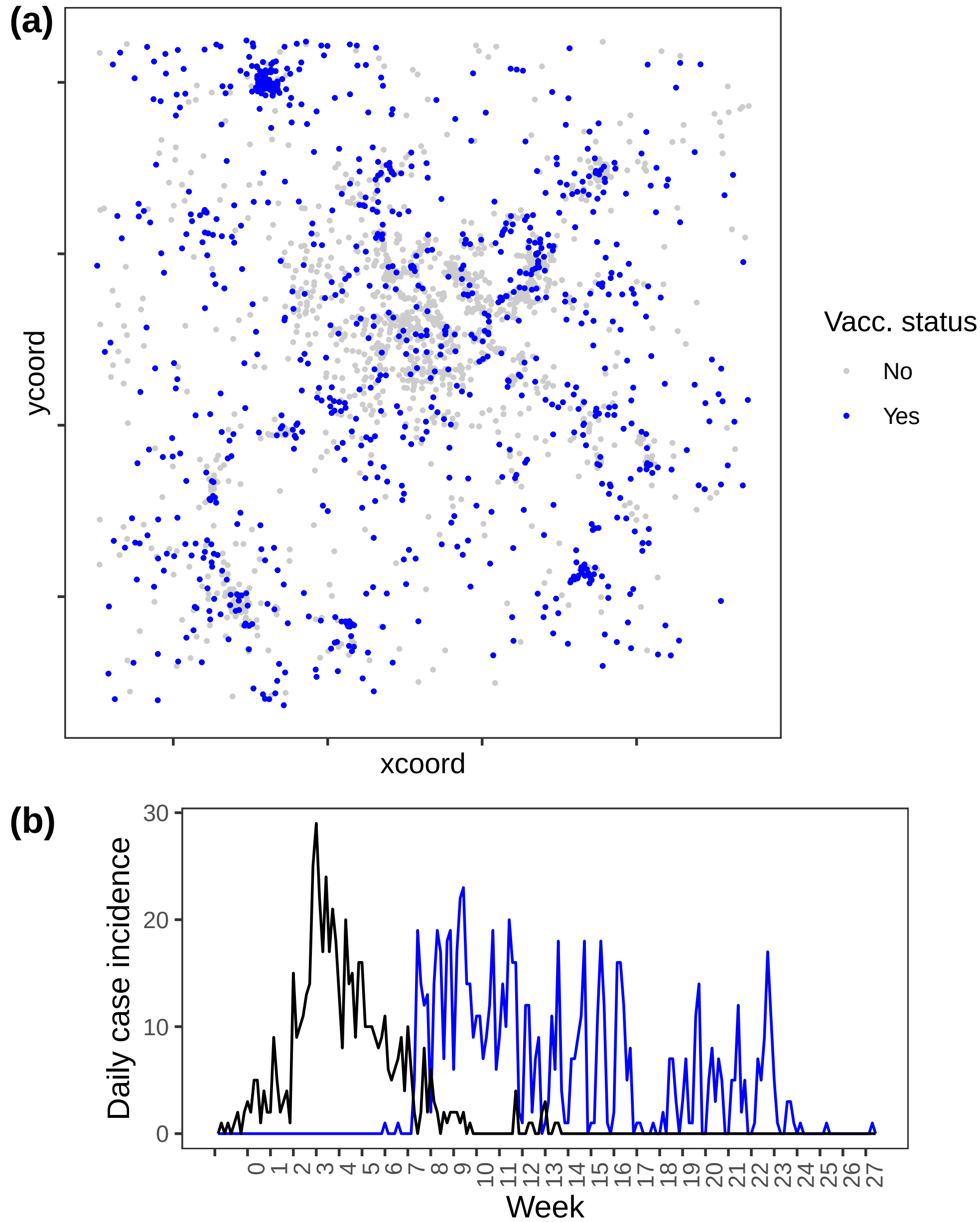

Fig B15: Vaccinations in the *Tamworth region*.

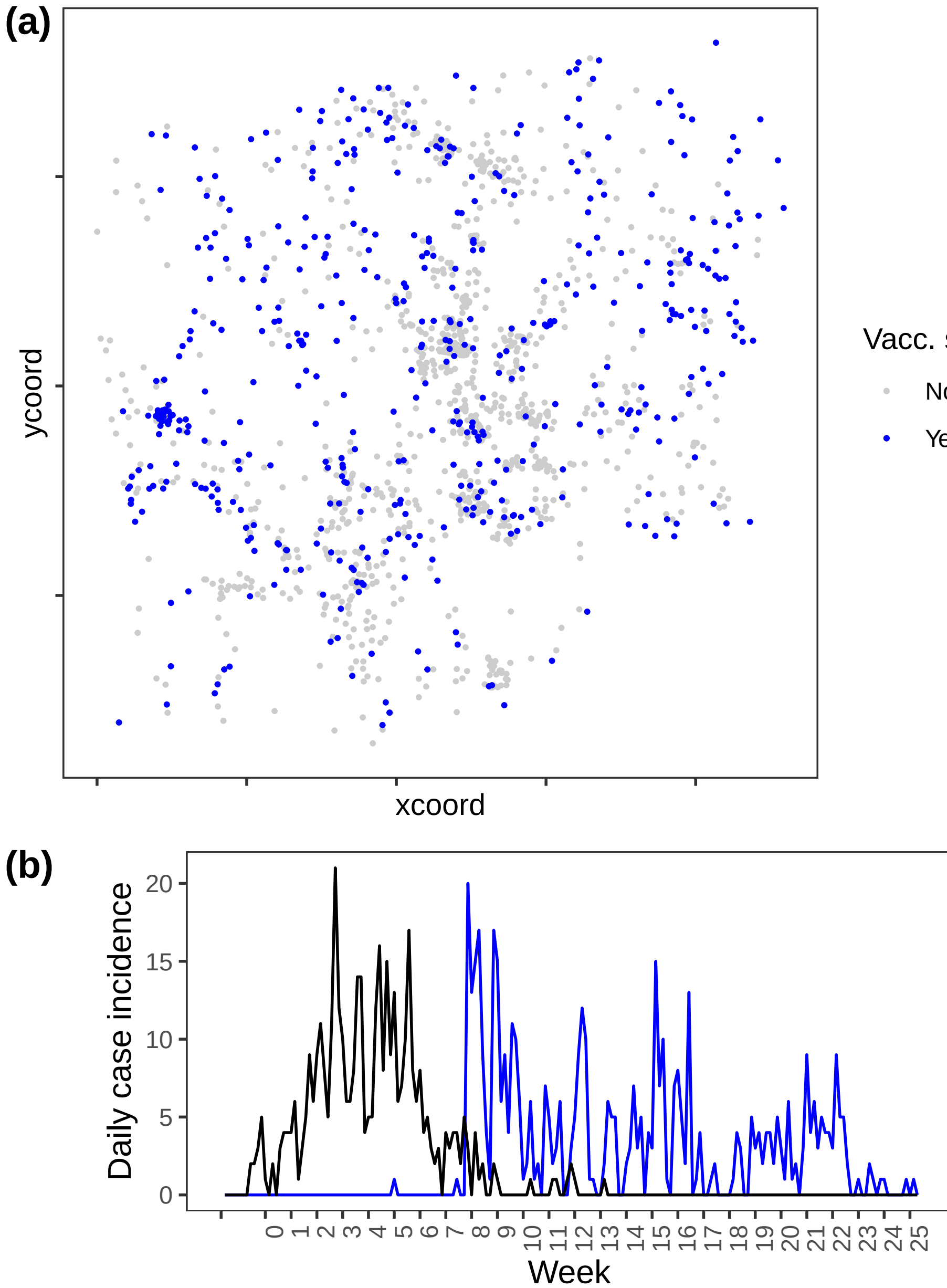

Fig B16: Vaccinations in the *Hunter Vallery region*.

References

Edlund Toulemonde, C., J. Daly, T. Sindle, P. M. Guigal, J. C. Audonnet, and J. M. Minke. 2005. "Efficacy of a Recombinant Equine Influenza Vaccine Against Challenge with an American Lineage H3N8 Influenza Virus Responsible for the 2003 Outbreak in the United Kingdom." *Veterinary Record* 156 (12): 367–71. https://doi.org/https://doi.org/10.1136/vr.156.12.367.

Firestone, Simon M. 2012. "Epidemiological Investigations into the 2007 Outbreak of Equine Influenza in Australia." PhD thesis, The University of Sydney.

Garner, M. G., B. Cowled, I. J. East, B. J. Moloney, and N. Y. Kung. 2011. "Evaluating the Effectiveness of Early Vaccination in the Control and Eradication of Equine Influenza—a Modelling Approach." *Preventive Veterinary Medicine* 99 (1): 15–27. https://doi.org/https://doi.org/10.1016/j.prevetmed.2010.02.007.

Minter, Amanda, and Renata Retkute. 2019. "Approximate Bayesian Computation for Infectious Disease Modelling." *Epidemics* 29: 100368. https://doi.org/10.1016/j.epidem.2019.100368.

Probert, Chris P. AND Werkman, William J. M. AND Jewell. 2018. "Real-Time Decision-Making During Emergency Disease Outbreaks." *PLOS Computational Biology* 14 (7): 1–18. https://doi.org/10.1371/journal.pcbi.1006202.
